# QSAR-Based Computational Approaches to Accelerate the Discovery of Sigma-2 Receptor (S2R) Ligands as Therapeutic Drugs

**DOI:** 10.3390/molecules26175270

**Published:** 2021-08-30

**Authors:** Yangxi Yu, Hiep Dong, Youyi Peng, William J. Welsh

**Affiliations:** 1 Department of Pharmacology, Robert Wood Johnson Medical School, Rutgers, The State University of New Jersey, Piscataway, NJ 08854, USA; yuyangxi@yeah.net; 2 Department of Medicinal Chemistry, Ernest Mario School of Pharmacy, Rutgers, The State University of New Jersey, Piscataway, NJ 08854, USA; hqd3@scarletmail.rutgers.edu; 3 Biomedical Informatics Shared Resource, Rutgers Cancer Institute of New Jersey, Rutgers, The State University of New Jersey, New Brunswick, NJ 08903, USA; pengyo@cinj.rutgers.edu

**Keywords:** Sigma-2 receptor (S2R), drug discovery, QSAR, pharmacophore model, optimization algorithms

## Abstract

S2R overexpression is associated with various forms of cancer as well as both neuropsychiatric disorders (e.g., schizophrenia) and neurodegenerative diseases (Alzheimer’s disease: AD). In the present study, three ligand-based methods (QSAR modeling, pharmacophore mapping, and shape-based screening) were implemented to select putative S2R ligands from the DrugBank library comprising 2000+ entries. Four separate optimization algorithms (i.e., stepwise regression, Lasso, genetic algorithm (GA), and a customized extension of GA called GreedGene) were adapted to select descriptors for the QSAR models. The subsequent biological evaluation of selected compounds revealed that three FDA-approved drugs for unrelated therapeutic indications exhibited sub-1 uM binding affinity for S2R. In particular, the antidepressant drug nefazodone elicited a S2R binding affinity Ki = 140 nM. A total of 159 unique S2R ligands were retrieved from 16 publications for model building, validation, and testing. To our best knowledge, the present report represents the first case to develop comprehensive QSAR models sourced by pooling and curating a large assemblage of structurally diverse S2R ligands, which should prove useful for identifying new drug leads and predicting their S2R binding affinity prior to the resource-demanding tasks of chemical synthesis and biological evaluation.

## 1. Introduction

The Sigma receptors (SRs), originally considered as members of the opioid receptor family [1], were then recognized as a separate group in 1982 [2]. There are two types of SRs: Sigma-1 receptor (S1R) and Sigma-2 receptor (S2R), which were first distinguished in 1990 [3]. The analgesic effects associated with modulation of S1R have been recognized [4], and more recent in vivo studies in rat models have demonstrated that S1R antagonists can potentiate opioid analgesia with fewer and less severe adverse effects such as drug tolerance [5]. Unlike S1R, S2R has attracted less attention. Initially in 2011, S2R was incorrectly identified as progesterone receptor membrane component 1 (PGRMC1) [6]. In 2015, this error was discovered using PGRMC1 knockdown experiments which revealed that the [3H^+^] DTG binding affinity is quite different between S2R and PGRMC1 [7]. In 2017, S2R was first reported as the translation of the transmembrane protein 97 gene (TMEM97) [8]. This conjecture was refuted in 2019, as knockout studies on TMEM97 and/or PGRMC1 demonstrated no effect on the EC50 of S2R ligands, suggesting that the cytotoxic effects of S2R ligands are not mediated by TMEM97 or PGRMC1 [9]. Moreover, unlike S1R, for which the human X-ray crystal structure is available (PDB ID: 5HK1), the crystal structure of S2R for any species remains unavailable.

S2R is known to play a functional role in cancer. In 1999, S1R and S2R were found to be implicated in prostate cancer, with potential to serve as both diagnostic biomarkers and therapeutic targets. In 2007, the dual S1R- and S2R-binding ligand PB183 was synthesized as a molecular probe for prostate cancer [10]. S2R upregulation was also found in various other malignant tumors, including lung cancer [11] and bladder cancer [12].

S2R overexpression is also associated with breast cancer. In 2001, a S2R ligand labeled with Tc-99m was found to be a potential breast tumor imaging agent [13]. The following year, a novel apoptotic pathway of S2R was found to potentiate antineoplastic drugs in breast cancer cell lines [14]. Moreover, a group of workers found that PGRMC1, a protein closely related to S2R, is a biomarker for the estrogen receptor in breast cancer [15]. Three years later, the relationship between breast cancer and S2R was established [6].

Between 2007 and 2012, Kashiwagi et al. [16] published a series of papers which showed that S2R preferentially binds to pancreatic adenocarcinomas, and S2R ligands improved efficacy when combined with pancreatic cancer chemotherapeutic drugs [17,18]. Their antitumor activity was found to be partly due to their induction of lysosomal membrane permeabilization [19].

The development of a S2R ligand as a tumor biomarker has entered clinical trials. The results of a phase 1 clinical trial (NCT00968656) published in 2013 demonstrated a high correlation between S2R ligand [^18^F]ISO-1 and Ki-67 (i.e., a common measure of the proliferative activity of breast cancer cells) in patients with breast cancer, head and neck cancer, and lymphoma, thereby indicating that [^18^F]ISO-1 shows promise for the evaluation of the proliferative status of solid tumors [20]. In another phase 1 clinical trial (NCT02284919) reported in 2020, [^18^F]ISO-1 demonstrated utility as a predictive biomarker for breast cancer proliferation [21].

Another target disease for S2R ligands is Alzheimer’s disease (AD). As proposed in 2014, S2R mediates Aβ-42 oligomer binding as well as synaptotoxicity, which indicate that S2R ligands may have potential as treatments for AD [22]. Later in 2017, researchers demonstrated the neuroprotective function of S2R ligands that can reduce cognitive deficits and neuroinflammation [23]. More recently, several clinical studies on the S2R antagonist CT1812 have been launched. A phase 1 clinical trial (NCT02570997: Ascending Dose Study of CT1812 in Healthy Volunteers) indicated satisfactory safety data [24]. Due to the prolonged observation period required to demonstrate AD treatment efficacy, as yet there are no available preliminary efficacy data for CT1812. However, according to ClinicalTrials.gov, the phase 2 study (NCT03507790: A Study to Evaluate the Safety and Efficacy of CT1812 in Subjects with Mild to Moderate Alzheimer’s disease) remains active as of the time when this manuscript was prepared. 

In the present study, we have employed computational tools to build distinct models that predict the biological activities of S2R ligands as potential therapeutic agents. We then implemented these models to virtually screen the DrugBank chemical database of existing drugs in search of S2R ligands that might be repurposed as treatments for serious diseases such as cancer and AD.

## 2. Results 

The 2D-QSAR and ligand-based pharmacophore models were constructed with different Ki datasets with overlap of some structures. The general workflow is shown in Figure S1.

### 2.1. 2D-QSAR

The X-ray crystal structure of S2R has not been published at the time this study was accomplished, thus ligand-based two-dimensional quantitative structure–activity relationship (2D-QSAR) models were developed and employed for virtual screening of selected chemical libraries to identify compounds with high S2R binding affinity. The 2D-QSAR models employed two dimensional (2D) descriptors which, by definition, are independent of the conformation of a molecule and are most suitable for large database studies.

A total of 159 non-redundant molecular structures from 16 publications met our selection criteria (Table 1). They were randomly split into a model building set of 127 structures and an external testing set of 32 structures. Considering the 127 entries in the modeling set, the ideal regression model should include no more than five descriptors according to Tropsha [25]. The modeling set was further randomly divided into a training set (102) and validation set (25). This process was repeated 50 times to minimize the risk of chance correlation. The linear regression models were built with the training sets and tested with the validation sets. After validation, with the descriptors determined, the 2D-QSAR model was built using the modeling set and tested using the external testing set.

Using the Molecular Operating Environment software (MOE 2018.08, Chemical Computing Group, Montreal, QC, Canada), each molecular structure was energy minimized after which chemical descriptors were generated. After removing certain descriptors that were deemed irrelevant to the activity, separate algorithms including stepwise regression (Appendix A), Lasso, genetic algorithm (GA), and an in-house developed extension of GA called GreedGene, were applied to select the descriptors and build separate QSAR models. The descriptors selected by each algorithm are listed in Table 2. The corresponding model training R^2^/Q^2^ and validation R^2^/Q^2^ are listed in Table 3.

**Figure 1:**
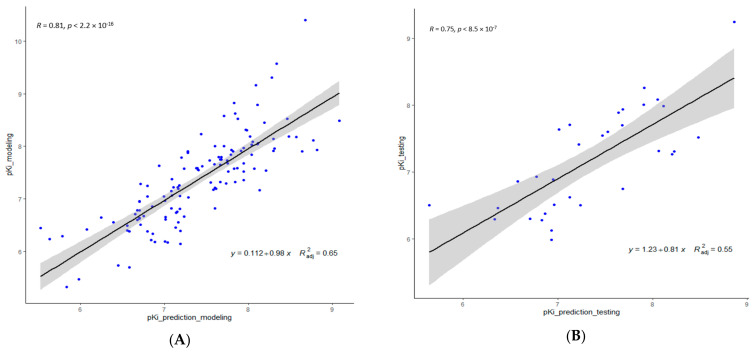
Linear regression plots of the 2D-QSAR model—predicted versus experimental pKi values of S2R ligands using the GreedGene descriptors: (**A**) modeling dataset; and (**B**) testing dataset.

Inspection of Table 3 reveals that both GA and GreedGene performed well with respect to their descriptor selection and the statistical performance of their corresponding QSAR model, while stepwise regression and Lasso showed comparatively poorer performance, which may be due to the bias caused by the most correlated independent descriptors selected by such algorithms. In comparison, due to additional exhaustive searching, GreedGene performs somewhat better than the typical GA. Consequently, we picked the descriptors selected by GreedGene to build our 2D-QSAR model plotted in Figure 1.

The 2D-QSAR model can be represented in analytical form by the following Equation (1):

pKi = 11.29 − 3.77 ∗ *balabanJ* + 0.23 ∗ *b_max1len* – 0.029 ∗ *Q_VSA_PNEG* + 0.043 ∗ *vsa_acc* − 0.026 ∗ *SlogP_VSA1* (1)

The five descriptors and their meanings are described below, viz., *balabanJ*, *b_max1len*, *Q_VSA_PNEG*, *vsa_acc*, and *SlogP_VSA1*. The *balabanJ* descriptor encodes information on the side chain of the molecules, which is a perfect addition to those descriptors concerning the main chain, such as *b_max1len*. The *b_max1len* descriptor represents the length of the longest single-bond chain, which reflects the conformational flexibility of the molecule and, by logical extension, to its ability to accommodate the receptor binding pocket. *Q_VSA_PNEG* encodes for the total negative polar van der Waals surface area (Å^2^) of the ligand in its charge-neutral (i.e., uncharged) state. Since all S2R ligands are characterized by at least one basic N atom, the value of this descriptor trends with the overall basicity of the S2R ligand and, specifically, with its ability to bind as an N-protonated species within the S2R binding pocket. The *vsa_acc* descriptor, which encodes for the van der Waals acceptor surface area (Å^2^) of pure hydrogen-bond acceptors, bears a positive correlation with pKi. Although the structure of the S2R binding pocket is unknown, we can infer that according to this descriptor there is at least one hydrogen-bond donor in the binding pocket to stabilize ligand binding. Finally, *SlogP_VSA1* encodes for the total van der Waals surface area occupied by atoms whose oil/water contribution (log P(o/w)) falls within the range −0.4 and −0.2. With *SlogP_VSA1* information, polarity information and hydrogen-bond acceptor information, it is possible to predict the bioactivity of certain S2R ligands. 

### 2.2. Pharmacophore Model

**Figure 2:**
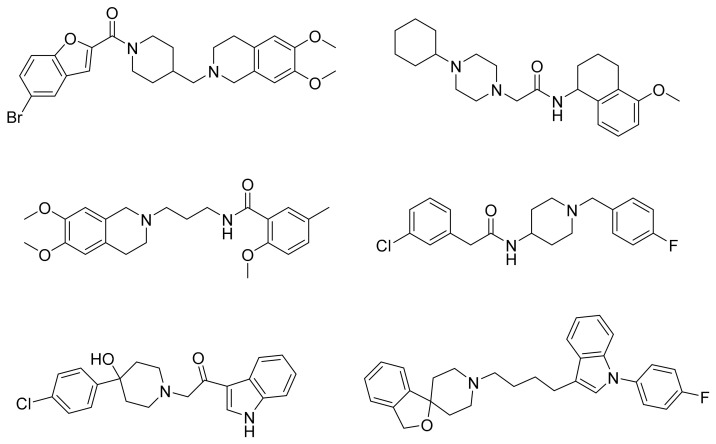
The six structurally diverse S2R ligands that were employed to construct the pharmacophore model.

Ligand-based pharmacophore modeling was employed using the Phase application in the Schrödinger software (ver. 2021; Schrödinger LLC, New York, NY, USA) [42]. Dataset P1 with six known S2R ligands was selected as the training set (Figure 2). These ligands were chosen based on their high S2R affinity and structural diversity to capture the most important pharmacophoric features. To avoid overweighting of any single family, one representative compound of each family was selected.

Different conformers were generated using MacroModel 9.7. S2R ligands with rigid structures were prioritized for inclusion in the training set, and a total of 948 conformers were generated for the training set. The superimposition process was implemented for these conformers with Phase. Given the absence of structural information for the S2R binding pocket, the excluded volume step was omitted to avoid false negative results. Consequently, the top 10 Phase Hypo Score models were retrieved using a scoring function chosen to quantify and compare the quality of each of the pharmacophore models. Generally, all models comprised five or six pharmacophoric points and shared three key features: one basic N atom, one aromatic ring, and one hydrophobic group. There is no model featuring more than one hydrogen-bond donor or acceptor group, consistent with the hydrophobic nature of the S2R ligand binding site.

To assess the quality of the pharmacophore models, 191 active S2R ligands were pooled from the literature with pKi cutoff of 6.0 (dataset P2). These compounds belong to three major scaffolds of S2R ligands, including siramesine analogs [43], piperazine [31,39,44,45,46,47], and tetrahydroisoquinolinyl [31,35,48,49,50,51,52,53]. Another core scaffold of S2R analogs, a conformationally restricted amine, was excluded from this study due to its high molecular weight and highly complex structure [54]. A set of decoys was generated based on these compounds via the DUD-E server (dude.docking.org), which is commonly used to evaluate the performance of virtual screening methods. Details on the selection and generation of these decoys is described in the Methods section. Dataset P3 (consisting of P2 and decoys) were virtually screened using each of the 10 pharmacophore models. A compound was considered “active” if it matched at least four out of five (or five out of six) of the pharmacophore features. From these results, statistical parameters were calculated and utilized as numerical criteria for comparing the quality of these pharmacophore models (Table 4).

**Figure 3:**
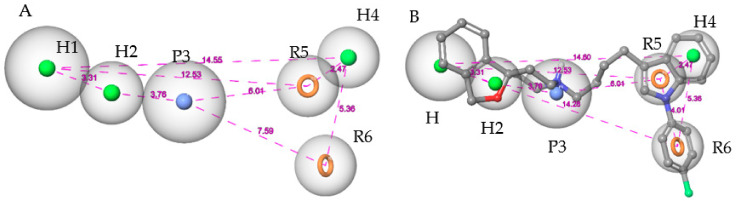
(**A**) The adjusted pharmacophore model. (**B**) Pharmacophore model with siramesine mapped inside. The spatial distances between the pharmacophoric elements are shown to emphasize the three-dimensionality of the pharmacophore model.

The Hypo 1 pharmacophore model outperformed all other models in terms of most statistical criteria, which was as expected given its high Hypo score derived by Phase. As depicted in Figure 3A, Hypo 1 comprises six pharmacophoric features, including three hydrophobic groups (H1, H2, H4), two aromatic groups (R5, R6), and one positively charged group (P3). Hypo 1 successfully discriminated the S2R ligands in the test set from the decoys with 80% sensitivity (152/191 true positives) and 96% specificity, attaining an enrichment factor (EF) of 15.2. To further optimize Hypo 1, the feature matching tolerance of H2, H4, R5, and R6 was calibrated to 1.5 Å while two other features, H1 and P3, were kept at the default value of 2.0 Å. This adjustment excluded additional decoys previously misidentified as “active” while maintaining the sensitivity of the model. It also improved the EF from 15.2 to 20.4. Mapping siramesine on the Hypo 1 model is illustrated in Figure 3B.

### 2.3. Shape-Based Screening

**Figure 4:**
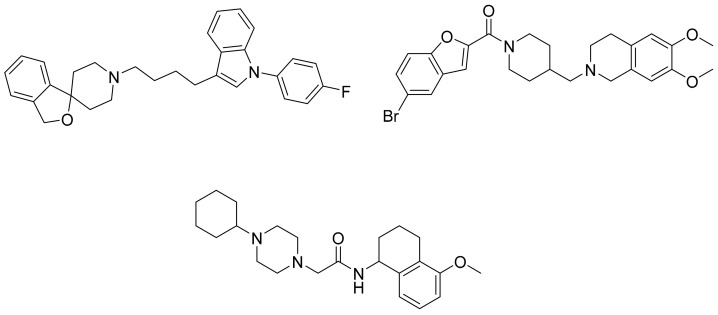
Three representative structures were selected as queries based on their high S2R binding affinity and conformational rigidity.

As an efficient high-throughput method, shape-based screening has been applied ubiquitously in virtual screening campaigns [55,56,57,58,59,60,61]. It has been recognized not only for outperforming structure-based docking methods in comparison studies [62] but also for excelling in scaffold hopping [63]. For shape-based methods to succeed, choosing the seed compounds and their conformers is a critical step [64]. In this study, three queries were chosen as representative compounds for the corresponding three core scaffolds of S2R ligands, including siramesine analogs, piperazine, and tetrahydroisoquinolinyl (Figure 4). They were selected based on their high binding affinity for S2R and their structural rigidity so to minimize the number of conformers. Given the absence of structural information for the S2R–ligand complex, energy-minimized conformers were generated and applied as structural queries for ligand-based virtual screening [64]. Initially, the shape-based search was run on dataset P3 to validate the protocol and to gauge our confidence level in future virtual screening workflows. The EF was calculated for the top 1% of the hit list for each seed compound. As a result, Compound 2 was excluded from the list of queries due to its poor performance.

### 2.4. Virtual Screening Workflow and Experimental Assay

Drug repurposing is an intriguing approach to reduce time, costs, and risk in developing new drugs. It offers significant advantages over traditional drug development [65,66]. The DrugBank database [67], which contains over 2000 FDA-approved drugs, was selected for the present study. The complete virtual screening cascade is depicted in Figure S1, basically involving QSAR modeling, pharmacophore screening, and shape-based screening. For the QSAR screening step, the predicted binding affinity (pKi) of 5.5 was chosen as the cutoff threshold for putatively active compounds. As a result, 823 compounds out of 2000 compounds were retained for pharmacophore-based screening. Subsequently, only 120 compounds in the DrugBank database matched at least five out of six pharmacophoric features of the refined Hypo 1 model. The top 20 hits predicted by shape-based screening with each seed compound were retrieved for manual inspection, amounting to a total of 30 compounds. Among these, there were six overlapping compounds shared by hit lists from two queries. Their binding affinities to S2R initially were checked as to whether they have been identified previously from the ChEMBL database [68] and DrugMatrix database (https://ntp.niehs.nih.gov/data/drugmatrix/, 5 July 2020). Seven out of thirty-four compounds were confirmed as S2R ligands with binding affinities in the micromolar range. Encouraged by this result, six other compounds from the hit list were retrieved for biological evaluation of their human S2R binding affinity taking into consideration their commercial availability, structural diversity, and pharmacological profile in particular their affinity for other off-target receptors. 

Six of these drugs were acquired from chemical vendors for initial biological evaluation of their human S2R binding affinity at a concentration of 1 μM (Table 5). The S2R binding assay was conducted in duplicate on human Jurkat Clone E6-1 cells using [3H]-DTG (0.025 μM) as the radioligand according to a previously reported protocol [69]. Nefazodone, cinacalcet, and pimozide exhibited 76%, 50%, and 70% binding affinity to the human S2R, respectively. On the other hand, ranolazine, flibanserin, and vilazodone showed 13%, 13%, and 26% binding affinity to the human S2R that fall below our selected “active” cutoff value (i.e., 50% inhibition at 1 μM). Notably ranolazine, as a racemic compound, falls outside of the applicability domains of the present model, which may explain its false positive prediction as a S2R ligand. A follow-up assay was then run for nefazodone, cinacalcet, and pimozide to determine their S2R binding affinity (Ki). Remarkably, they demonstrated potent binding affinity for the human S2R with Ki = 140 nM (nefazodone), Ki = 490 nM (cinacalcet), and Ki = 400 nM (pimozide). 

## 3. Discussion and Conclusions

S2R overexpression is associated with multiple life-threatening pathologies, including various forms of cancer (e.g., breast, prostate, lung, bladder, pancreas, skin, and ovary). It has also been shown to play a role in both neuropsychiatric disorders (e.g., schizophrenia) and neurodegenerative diseases, particularly Alzheimer’s disease (AD). In the present study, three ligand-based methods, viz., QSAR modeling, pharmacophore mapping, and shape-based screening, were employed to extract a subset of putative S2R ligands from the DrugBank library composed of 2000+ entries. Subsequent biological evaluation of six of these compounds yielded encouraging results, i.e., three FDA-approved drugs for unrelated therapeutic indications exhibited sub-1 uM binding affinity for S2R. In particular, the antidepressant drug nefazodone elicited a binding affinity Ki = 140 nM for S2R. Therefore, nefazodone would serve as an excellent starting point for a drug discovery campaign aimed at the rational design and optimization of S2R-mediated therapeutics for cancer, AD, and other serious diseases.

A total of 159 S2R ligands that met our selection criteria were retrieved from 16 publications. They were randomly divided into two sets, one for model building (127 structures) and another for model validation and external testing (32 structures). Multiple linear regression models were constructed and tested to predict as-yet-unknown S2R ligands. Four separate optimization algorithms (i.e., stepwise regression, Lasso, genetic algorithm (GA), and a customized extension of GA called GreedGene) were applied to select the most information-rich chemical descriptors and to construct and implement our QSAR models.

In conclusion, we believe that the 2D-QSAR models developed here for virtual screening of large databases to predict S2R ligands are the first of their kind and were constructed from a large (159 compounds) and structurally diverse set of compounds. Hypo 1 is the first pharmacophore model of S2R ligands which was constructed from a structurally diverse set of compounds. Laurini et al. [69] developed a five-featured pharmacophore model from a single series of benzo[d]oxazol-2(3H)-one derivatives. Striking differences between Hypo 1 and the model developed by Laurini et al. are found in the number of pharmacophoric features, the absence of a hydrogen-bond acceptor group, and the spatial arrangement. Additionally, the fact that the majority of known active S2R ligands in the test set match the Hypo1 model suggests that S2R ligands of these three main scaffolds may share the same active site, similar spatial orientation, and drug–receptor interactions with S2R. Encouraged by this observation, the adjusted Hypo 1 model was selected to use in the virtual screening workflow for novel S2R ligands. We are hopeful that our present efforts will inspire the biomedical research community to further explore the structure and biological function of S2R under both normal and pathological conditions. Moreover, we anticipate that the computational virtual screening approaches employed here will stimulate the rational design of therapeutics for serious S2R-mediated pathologies, including cancer, schizophrenia, and AD. Such is the case here with the existing drug nefazodone that may be repurposed to serve as a starting point for a drug discovery campaign. 

## 4. Materials and Methods

### 4.1. Data Preparation

#### 4.1.1. QSAR Data

S2R ligands were retrieved from a defined subset of literature published in 2000 or later, and publications with 3 or fewer applicable compounds were excluded. The data collected from these publications were required to have used the same radioligand ([3H^+^] DTG) and the same cell species (rat liver membrane cells). The selected S2R ligands met the following requirements: (i) molecular weight below 500 g/mol; (ii) binding affinity (Ki value) less than 5000 nM (5 uM); (iii) exclude compounds with the scaffold 9-azabicyclo (3,3,1) nonane, which is significantly different from known S2R ligands; (iv) require a basic nitrogen atom embedded in a rigid cyclohexyl ring; and (v) exclude structures with one or more chiral centers unspecified with respect to their exact stereospecificity. Structures and activity data of all S2R ligands can be found in the Supplementary Materials.

The molecular structures and corresponding pKi values were pooled and entered into Molecular Operating Environment (MOE) version 2018.01. The QSAR model generation followed best practices as recommended by Tropsha [25]. Each S2R ligand structure was curated and represented in its protonated form at pH 7 enumerated using the Wash function. The energy minimization was performed using the MMFF94 force field with the gradient cutoff at 0.001 RMS kcal/mol/A^2^.

#### 4.1.2. Pharmacophore Data Collection

The initial database of 197 non-redundant S2R ligands was divided into two subsets, designated dataset P1 and dataset P2, for subsequent generation of a pharmacophore. Dataset P1 comprised 6 S2R ligands, employed as the training set to identify common pharmacophoric features. Dataset P2 comprised 191 S2R ligands to evaluate the performance of pharmacophore models. A pKi cutoff of 6 was chosen corresponding to S2R ligands with binding affinities at least in the sub-micromolar range. To assess the ability of pharmacophore models to distinguish S2R active from inactive compounds, a set of decoys was generated from the structural information of compounds in dataset P2 via the DUD-E server [70]. The Tanimoto coefficient (Tc) [71] of the decoys to any ligand was calculated, and the maximum Tc was employed to sort potential decoys. The most dissimilar 25% based on their Tc value were utilized as the cutoff to yield the set of decoys. The optimal ratio of actives and inactives should be 1:50. This scheme was employed to generate 12,148 decoys defined as “inactives” which, together with the 191 active S2R ligands in dataset P2, yielded dataset P3 comprising 12,339 compounds.

All compounds in the datasets were prepared using the LigPrep application in Maestro 11.2 and protonated at the basic nitrogen atom at pH = 7.4, since this feature is required for all high affinity S2R ligands. Finally, molecular mechanics energy minimization was performed using the OPLS3 force field [72] with convergence thresholds set to their default values.

Conformers of each ligand were generated with MacroModel 9.7 implemented in Maestro, applying the OPLS3 force field. The generalized Born/surface area (GB/SA) solvation model was utilized to simulate solvation effects, with no cutoff value set for non-bonded interactions [73]. Then, the Polak–Ribiere conjugate gradient (PGCG) method was employed for energy minimization with gradient convergence thresholds of 0.001 kJ/mol/Å and 2500 maximum iterations. Monte Carlo multiple minimum (MCMM) torsional sampling was used to conduct the conformational search. Default values were applied for the cutoff of maximum atom deviation and for the energy window to save structures and to eliminate redundant conformers.

### 4.2. Data Splitting

By exporting the molecular attributes data in csv format and processing by the program R 3.3.3 [74], these molecules were randomly split into a modeling set and validation set (external testing set) corresponding to a ratio of 4:1. The modeling set was randomly split into a training set and testing set at a ratio of 4:1 for 50 times initialized using different random seeds. As the result, 50 instances of training set and testing set were generated. The modeling process was implemented for each of 50 different splits in order to minimize chance correlation.

#### 4.2.1. Descriptor Selection

For building the 2D-QSAR models, we limited our choices to two-dimensional (2D) descriptors that are conformation independent. A total of 206 2D descriptors were calculated. Moreover, values of the experimental binding affinity Ki were converted to pKi following the equation pKi = 9 − log (Ki) using the calculation function in MOE.

#### 4.2.2. Descriptor Screening

As there were 127 molecules in our modeling set, and best practices specify a model with 5 or fewer descriptors corresponding to a ratio of 1:25. The initial set of molecular descriptors was screened to exclude certain types: First, descriptors deemed to lack clear physical meaning or relationship to the binding affinity were excluded. For example, “rsynth”, which represents the difficulty for synthesis of the compound, was excluded based on its lack of relevancy of this descriptor to the S2R ligand. Then, descriptors that gave the value of 0 in most of the molecules were excluded since it is unlikely that such descriptors are capable of distinguishing differences in the binding affinity (pKi) among the molecules. For example, the descriptor “SlogP_VSA6” gives a value “0” in over 95% of the molecules and thus was excluded. In addition, descriptors that give values that are distributed as binary numbers were excluded since they would obviously decrease the accuracy of our model. For example, “vsa_don”, even though “0” is not the majority, it was still excluded because the value is either “0” or “5.6826”.

#### 4.2.3. Advanced Descriptor Selection: Lasso, Stepwise, and Lars

Developed by Robert Tibshirani in 1996, Lasso [75] introduces a penalty value lambda (λ) into the equation of the linear regression model as a limitation to the coefficient. Compared with the original least-squares method, this limitation provided criteria to reduce the probability of overfitting. Compared with Ridge regression, which forms a curved region for which it is difficult to provide an intersection on an axis, Lasso can help select a limited number of features provided by the linear (straight) region, which is more likely to have the axes intersect. Intersection on a certain axis represents the nil (0) value of some coefficient and, thus, the number of descriptors is reduced.

Stepwise regression is another method for feature selection. Here, we used the forward stepwise method, which determines the most correlated descriptor as the starting point, then combines this descriptor with each of the other descriptors to obtain the best two descriptors and continues this process recursively to get three or more descriptors. This algorithm is regarded as a type of theoretically unstable “greedy” strategy, but it has been found to be quick and effective in many cases.

To circumvent problems encountered sometimes with stepwise and Lasso, the Lars (least angle regression) algorithm was applied [76]. The algorithm is similar to forward stepwise regression, but instead of including variables at each step, the estimated parameters are increased in a direction equiangular to each one’s correlations with the residual. This is a recursive process, and the operator can choose a stop point at each step. All the methods above are performed with Lars package in R 3.3.3.

### 4.3. Genetic Algorithm (GA)

The GA algorithm designed by J. H. Holland mimics the process of natural selection [77]. In this algorithm, each descriptor is considered as a gene which adopts the value 0 or 1 to represent its absence or presence, respectively. RapidMiner 8.0 was employed to perform the GA for this study. The advantage of this program is that every module is visualized and, like objective coding software, can be easily integrated with each module to complete an entire process. Before GA selection, we removed the correlated descriptors between which the correlation is over 0.9. Such descriptors may overweight the contribution of certain descriptors and make the model inaccurate. The resulting data are used for GA selection.

### 4.4. GreedGene

Traditional algorithms (aside from GA) tend to select the most correlated descriptor as the first step. Based on this descriptor, the other descriptors are added in receding order of importance (weight). However, the most correlated descriptor may contain redundant information which may interfere with the accuracy of our prediction. GA is quite different since the starting point as it is completely random. By crossover with each other, the result will be improved in each generation at early stages, from where we can achieve a comparatively optimal result.

After a certain number of generations, the populations in GA tend to be stable, which means that extending for extra generations will no longer produce any significant improvement on the outcome. To overcome this limitation of the GA, we modified the GA by integrating it with what is known as the exhaustive algorithm, thereby coined as “GreedGene”. We designed it to particularly deal with cases like ours: with a single digit number of descriptors and hundreds of molecules. The concept of GreedGene is to take advantage of GA’s efficiency in the early stage and use the so-called exhaustive algorithm to avoid the weakness of GA in the later stage. By repeating GA several times, in those groups of descriptors with high performance, some descriptors were commonly seen in all or most of those groups, which we considered as important in a model. These descriptors thus selected are considered as “synergistic (complementary)” descriptors. The term “synergistic” in this context means that these descriptors cooperate with other descriptors better and provide us with a more accurate model in general. Based on these descriptors, an exhaustive search is performed to select the remaining descriptors.

The exhaustive algorithm is the simplest and most basic but most accurate and time consuming one. The principle behind this algorithm is to try every possible combination of descriptors. The theoretical timing for our case is C (160,5) = 820,384,032 times of regression model building, which exceeds the capacity of a personal computer to calculate in a reasonable amount of time. As an estimation, 1 million cycles of regression model building with R 3.3.3 takes 2 h for a 7th generation of a Core i5 processor. Extending this to 820 million cycles would require 1640 h or approximately 68 full days. Furthermore, the memory requirement for this case is also unrealistic for most computers to process.

The principle behind our new algorithm is that by repeating genetic selection with RapidMiner 8.0 several times using different random seeds, we can generate several different groups of descriptors. By comparing these groups, those descriptors commonly seen in every group were considered as significant. With these significant descriptors so determined, we searched for additional descriptors by the exhaustive algorithm in R 3.3.3. Given the present case in which 2 of the 5 descriptors are already defined, we only need to find 3 additional common descriptors. Under these circumstances, exhaustive searching only requires 666,000 cycles of regression model building to select the 3 additional descriptors which is acceptable for most modern computers. For example, it takes a 7th generation i5 processor around 1.5 h in R 3.3.3 to complete this calculation. This algorithm is theoretically better than, or at least equivalent to, the GA, since the exhaustive process tried all possible combinations, including all those groups of descriptors we obtained from the GA. Compared with pure exhaustive search, this algorithm consumes significantly less time. In general, it reaches an acceptable balance between timing and accuracy.

Since we randomly chose one descriptor from all the correlated descriptors in GA selection, we double check the result by replacing each descriptor with its correlated ones, and the one with the best performance is kept. As this algorithm is based on the typical GA, and the design of such algorithm adopts the concept of greedy strategy, we named it GreedGene.

### 4.5. QSAR Model Generation and Validation

#### 4.5.1. Training

As we randomly split the modeling set 50 times, for each split training set, models were built with selected groups of descriptors employing multivariate linear regression (MLR) in R 3.3.3. In total we have 4 groups of descriptors, and all 4 groups were calculated for each split.

The training R^2^ (squared-correlation coefficient of the training set) was computed to evaluate the performance of modeling of each data split. In our case, the criterion for R^2^ is over 0.6. Repetitive splitting 50 times decreases chance correlation and, thus, the R^2^ range of all splits were calculated.

Q^2^ (squared-correlation coefficient for leave-one-out cross-validation) was also calculated for evaluating the quality of the model. This parameter removes one compound and uses the model built from the remaining molecules to predict the pKi value of the omitted one. A linear regression model was built, for which Q^2^ is 0.5 [78]. As for R^2^, the Q^2^ range of the splits was calculated. 

#### 4.5.2. Validation

With all the models built, for each data split, the corresponding testing set was applied to the model and the prediction made. According to the prediction and the true value, validation R^2^ is calculated. This parameter represents how this model predicts our validation set, with the criteria of 0.5. The range of testing R^2^ was calculated, and the percentage of splits that meet the criteria is calculated where 95% is considered as a stable model.

#### 4.5.3. Testing

Finally, each model was applied to the testing set, and the squared-correlation coefficient between the prediction value and the actual value (testing R^2^) was calculated. There was no strict criterion for this value, except that a higher value represents better performance. Training R^2^, Q^2^, testing R^2^, and validation R^2^ were all criteria for evaluating the performance of our models. The performance between all 4 groups of descriptors was compared and the best one was retained as our 2D-QSAR model.

### 4.6. Pharmacophore Hypothesis Generation and Evaluation 

The pharmacophore model generation was automatically conducted using the Phase application, implemented in Maestro [79]. In detail, all resulting conformers of S2R ligands in the dataset P1 were superimposed to identify the pharmacophore hypothesis (Hypo) including four to six features. A pharmacophore hypothesis was retained only if it matches at least 70% of active compounds in the training set. The hypothesis difference criterion was set at the default value 0.5. Furthermore, because of the importance of the basic nitrogen for S2R activity, there must be one positive ionic feature in the resulting pharmacophore hypothesis. The default scoring function, Phase Hypo Score, was implemented to rank the resultant pharmacophore hypotheses. No other constraint or excluded volume was set due to the lack of structural information of S2R. Eventually, the top 10 pharmacophore hypotheses (Hypo 1–10) ranging from five to six features were reported before proceeding to the evaluation process. 

For pharmacophore hypothesis generation, the evaluation of the pharmacophore model was carried out using the phase application [79]. Initially, the S2R ligands in the test set, dataset P3, were prepared using the same protocol as the compounds in the training set. A similar protocol of conformer generation as the training set was also applied for the testing set. Then, each pharmacophore hypothesis was alternately applied to the dataset P3. Compounds could partially match the pharmacophore hypothesis; however, they were required to fulfill at least four out of five (or five out of six) features which must additionally include the positive ionic feature (i.e., the protonated N atom). The *Sensitivity* (Equation (2)) and *Specificity* (Equation (3)) were calculated for each model. Furthermore, the *EF* (Equation (4) was considered to evaluate the quality of the pharmacophore hypothesis [80]. Subsequently, the top performing model was retained to employ in the virtual screening protocol to explore new S2R ligands.

(2) $$Sensitivity=\frac{\mathrm{TP}}{\mathrm{TP}+\mathrm{FN}}$$

(3) $$Specificity=\frac{\mathrm{TN}}{\mathrm{TN}+\mathrm{FP}}$$

(4) $$\mathrm{Enrichment}\;\mathrm{Factor}\;=\frac{\mathrm{TP}/\mathrm{Ht}}{A/D}$$

TP: number of true positives in the hit list. TN: number of true negatives in the hit list. FP: number of false positives in the hit list. FN: number of false negatives in the hit list. Ht: number of hits selected from the database. A: number of total active compounds in the database. D: total number of entries in the database

### 4.7. Shape-Based Screening 

As an efficient and high-throughput method, shape-based screening has been applied ubiquitously in virtual screening campaigns. The three most active S2R ligands, representing three major scaffolds of S2R ligands, were designated as queries for shape-based screening. Several publications of shape-based screening have pointed out that in cases where a co-crystal ligand is lacking, the energy-minimized structures should be used as the query to achieve the optimal enrichment rate [64]. Consequently, the energy-minimized conformer of each solvated ligand in water was retrieved by MacroModel 9.7 using the OPLS3 force field. Specifically, the Powell–Reeves conjugate gradient algorithm was applied with convergence threshold of 0.001 kJ/mol/Å and maximum iteration of 2000. Then, to validate the chosen protocol, three queries were run on dataset P3. As the default setting, up to 10 conformers were retained per rotatable bond and amide bonds were kept in their original conformation. The MacroModel atom type was adapted for volume scoring and Shape Sim Score calculation to consider the contribution of the entire molecule instead of pharmacophore features only [81]. The EF of the top 20 compounds, ranked by Shape Sim Score, was calculated for each query. Consequently, compound 2 was removed from the list of queries because of its poor performance. 

### 4.8. Virtual Screening Protocol 

A protocol of virtual screening cascade was applied on the DrugBank database [68,82], which contained a total of 2334 approved drugs. Prior to the prediction, peptidomimetic compounds, inorganic compounds, and drugs which violated Lipinski’s Rule of Five (RO5) [83] were excluded. Furthermore, the same criteria as applied in the QSAR modeling set were employed to filter out compounds which may be outside of the applicability domain of the QSAR model. For instance, compounds without a basic N atom or more than two positive charged groups were removed from the database. Molecules passing the filtering step were prepared using the protocol mentioned above. Using the molecular descriptors computed in MOE, their predicted pKi values for S2R were computed by the 2D-QSAR model. The pKi of 5.5 was chosen as the cutoff, resulting in a subset of 834 compounds for the subsequent screening steps. 

The 834 compounds prioritized by the 2D-QSAR model were prepared using LigPrep as the protocol applied for datasets P1 and P3. Subsequently, the chosen pharmacophore model was applied on these compounds, preceded by conformer generation. As a result, the process resulted in 120 putative S2R ligands which matched the pharmacophore hypothesis. In the last round of the virtual screening cascade, shape-based screening with two queries was implemented. Of the top 20 compounds of each query, six compounds were manually selected for biological evaluation of their biological activities and potential to be repurposed for new therapeutic indications.

### 4.9. Radioligand Binding Assay

The bioassay was performed generally according to the protocol published previously [69]. The human Jurkat cell line Clone E6-1 cells were incubated in RPMI 1640 medium with 10% FBS, 100 U/mL penicillin and 100 mg/mL streptomycin. After incubation, the cells were suspended in 5 mM potassium phosphate buffer (pH = 7.6). The membrane protein concentration was controlled at 5 mg/mL.

The collected membrane protein was incubated with ligands in potassium phosphate buffer for 1 h at room temperature and terminated by adding ice-cold buffer. Finally, the mixture was filtered with 0.7 µm syringe filter and washed three times. The radioactivity is determined by liquid scintillation spectrometry. The S2R binding assay was performed with 0.025 µM [3H^+^] DTG as the radioligand. The non-specific binding was performed with 10 µM haloperidol.

## Figures and Tables

**Table 1:** Summary of the 159 S2R-active compounds, including their generic structure, number of compounds, and published source, compiled for the QSAR modeling.

ID	Reference	pKi Range	Number of Compounds	Structure
1	Ferorelli, Abate [26]	5.48–7.71	9	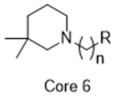
2	Mach, Huang [27]	6.14–8.09	8	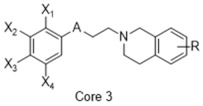
3	Huang, Luedtke [28]	6.39–6.95	4	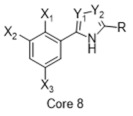
4	Mach, Huang [29]	6.29–7.59	9	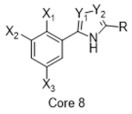
5	Yarim, Koksal [30]	6.18–8.00	6	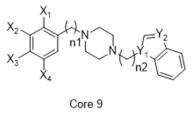
6	Abate, Ferorelli [31]	6.51–8.79	14	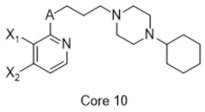
7	Niso, Abate [32]	7.54–10.40	9	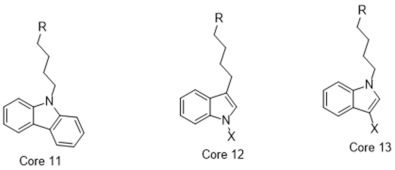
8	Abate, Ferorelli [33]	7.29–8.58	8	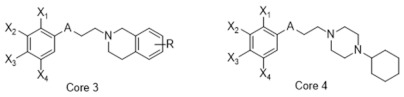
9	Berardi, Ferorelli [34]	7.52–9.24	4	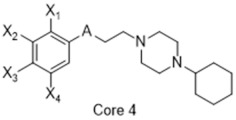
10	Bai, Li [35]	5.99–8.82	22	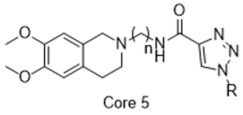
11	Xie, Bergmann [36]	6.28–7.64	16	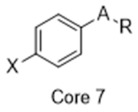
12	Berardi, Ferorelli [37]	6.62–7.75	15	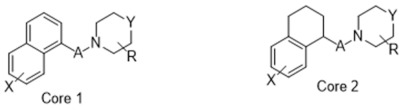
13	Ferorelli, Abate [38]	6.17–8.08	8	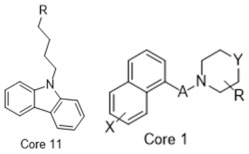
14	Abate, Niso [39]	7.63–9.31	7	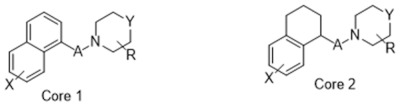
15	Xie, Kniess [40]	7.17–8.52	10	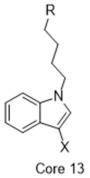
16	Schininà, Martorana [41]	5.33–7.25	10	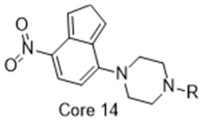

**Table 2:** Sequence of top five chemical descriptors selected by each algorithm.

**Lasso**	b_Single	Chi0v_C	Chi1v_C	b_max1len	QRPC +
**Stepwise**	b_single	chi1_C	SMR_VSA2	BCUT_PEOE_3	SlogP_VSA9
**GA**	balabanJ	b_max1len	SMR_VSA0	Q_VSA_FPNEG	SMR_VSA3
**GreedGene**	balabanJ	b_max1len	Q_VSA_PNEG	vsa_acc	SlogP_VSA1

**Table 3:** List of statistical parameters calculated for each QSAR model using the separate optimization algorithms.

Statistical Parameters	Lasso	Stepwise	GA	GreedGene
Training R^2^	0.43–0.58	0.48–0.60	0.58–0.68	0.62–0.69
Training Q^2^	0.36–0.52	0.42–0.56	0.52–0.63	0.57–0.64
Validation R^2^	0.27–0.68	0.37–0.71	0.50–0.73	0.53–0.78
% met criteria	38%	68%	100%	100%
Modeling R^2^	0.5	0.55	0.63	0.65
Modeling Q^2^	0.45	0.50	0.59	0.62
Testing R^2^	0.51	0.51	0.51	0.56
Criteria met	Yes	Yes	Yes	Yes

**Table 4:** Summary of the 10 best pharmacophore models (i.e., Hypo 1–10).

Hypo 1	HHHPRR	15.2 *
Hypo 2	HHPRD	7.8
Hypo 3	HDPRR	6.4
Hypo 4	HDPRR	4.5
Hypo 5	HAPRR	3.2
Hypo 6	HHPRDH	5.2
Hypo 7	HAPRR	3.7
Hypo 8	AHPRR	2.1
Hypo 9	AHPRR	4.1
Hypo 10	HHPRR	4.3

* EF = Enrichment Factor.

**Table 5:** Hits from virtual screening of the DrugBank database with results from human S2R binding assays *.

Generic Name	Structure	Inh% at 1 μM
Ranolazine	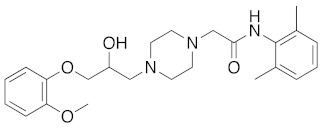	13
Flibanserin	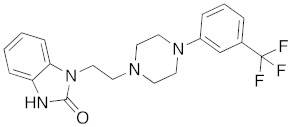	13
Nefazodone	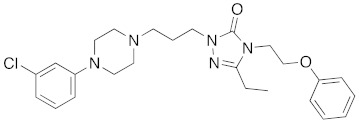	76
Cinacalcet	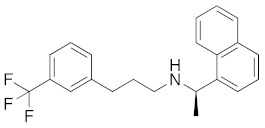	50
Pimozide	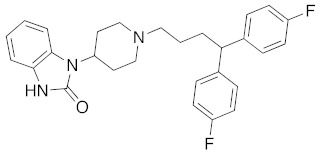	55
Vilazodone	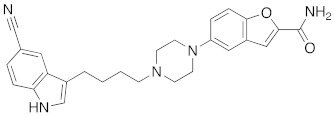	26

* Binding assays were performed by Eurofins Panlabs Discovery Services.

## Data Availability

The DrugBank database can be found at https://www.drugbank.com/ (accessed on 21 August 2021).

## Supplementary Materials

The following are available online. Figure S1: Virtual screening cascade is depicted in Figure 1, basically involving QSAR modeling, pharmacophore screening, and shape-based screening. Table S1: Structures and activity data of all S2R ligands pooled from different sources for the present 2D-QSAR studies. Table S2: Summary of the specific scaffold (core), nature of the substituents X, Y, A, etc., values of the experimental and QSAR model-predicted pKi, the Residual (Res.) = Exp. pKi – Pred. pKi, and the identification of the dataset. Table S3: SMILES structure for all S2R ligands used in the present study.

## Sample Availability

Samples of the compounds are available from the authors.

## Appendix A

**R codes**

**Data splitting**

a = read.csv(“159.csv”, header = T)

sample <- sample.int(n = nrow(a), size = floor(.8*nrow(a)), replace = F)

int = a[sample,]

ext = a[-sample,]

write.csv (int,”in159.csv”)

write.csv (ext,”ex159.csv”)

**Stepwise regression**

b = read.csv(“in159.csv”, header = T)

d = scale(b)

x = as.matrix(d[, 3:162])

y = as.matrix(d[, 2])

library(lars)

la = lars(x,y,type = “stepwise”)

plot(la)

summary(la)

la

**Lasso regression**

b = read.csv(“in159.csv”, header = T)

d = scale(b)

x = as.matrix(d[, 3:162])

y = as.matrix(d[, 2])

library(lars)

la = lars(x,y,type = “lasso”)

plot(la)

summary(la)

la

**Code for GreedGene exhaustive phase**

b = read.csv(“in159.csv”, header = T)

d = scale(b)

x = as.matrix(d[, 3:162])

y = as.matrix(d[, 2])

t = 0

p = 0

q = 0

s = 0

for (j in 3:162)

for (j in i:162)

for (k in j:162)

{ r[i,j,k] = summary(lm(y~x[,i] + x[,j] + x[,k] + x[, 11] + x[, 30]))$adj.r.squared

if (r[i,j,k] > t){t = r[i,j,k]

p = i

q = j

s = k}

}

p

q

s

t

**Validation batch**

int = read.csv(“in159.csv”, header = T)

ext = read.csv(“ex159.csv”, header = T)

q = c(1:50)

dim(q) = c(50)

r = c(1:50)

dim(r) = c(50)

rm = c(1:50)

dim(rm) = c(50)

t = c(1:50)

dim(t) = c(50)

for (i in 1:50)

{set.seed(i)

sample1 <- sample.int(n = nrow(int), size = floor(.8*nrow(int)), replace = F)

train = int[sample1,]

test = int[-sample1,]

model = lm(pKi~balabanJ + b_max1len + Q_VSA_PNEG + vsa_acc + SlogP_VSA1, data = train)

y = as.matrix(train[, 1])

re = c(1:nrow(train))

dim(re) = c(nrow(train))

for (j in 1:(nrow(train)))

{trains = train[-j, ]

traink = train[j, ]

model2 = lm(pKi~balabanJ + b_max1len + Q_VSA_PNEG + vsa_acc + SlogP_VSA1, data = trains)

re[j] = predict(model2, traink)}

q[i] = summary(lm(y~re))$r.squared

r[i] = summary(model)$r.squared

tr = predict(model, test)

t[i] = summary(lm(test$pKi~tr))$r.squared}

model1 = lm(pKi~balabanJ + b_max1len + Q_VSA_PNEG + vsa_acc + SlogP_VSA1, data = int)

ppp = predict(model1, ext)

ccc = ext[,“pKi”]

summary(lm(ppp~ccc))

z = as.matrix(int[, 1])

rex = c(1:nrow(int))

dim(rex) = c(nrow(int))

for (j in 1:(nrow(int)))

{ints = int[-j, ]

intk = int[j, ]

model3 = lm(pKi~balabanJ + b_max1len + Q_VSA_PNEG + vsa_acc+SlogP_VSA1, data = ints)

rex[j] = predict(model3, intk)}

qq=summary(lm(z~rex))$r.squared

r

q

t

summary(model1)

qq

summary(lm(ppp~ccc))$r.squared
