# Supplementary material for: QSAR-Based Computational Approaches to Accelerate the Discovery of Sigma-2 Receptor (S2R) Ligands as Therapeutic Drugs
Source: Molecules. 2021 Aug 30;26(17):5270. doi: 10.3390/molecules26175270 (PMC8434483; doi:10.3390/molecules26175270)
Supplement: Supplementary file 1 [file molecules-26-05270-s001.zip › Molecules-1304403 Supplementary Materials - update system.pdf]

Supplementary Information File

QSAR-based Computational Approaches to Accelerate the Discovery of Sigma-2 Receptor (S2R) Ligands as Therapeutic Drugs

Yangxi Yu[1], Hiep Dong[2], Youyi Peng[3], William J. Welsh[1] *

1 Department of Pharmacology, Robert Wood Johnson Medical School, Rutgers, The State University of New Jersey, Piscataway, NJ 08854, United States; yuyangxi@yeah.net, welshwj@rwjms.rutgers.edu

2 Department of Medicinal Chemistry, Ernest Mario School of Pharmacy, Rutgers, The State University of New Jersey, Piscataway, New Jersey 08854, United States. hqd3@scarletmail.rutgers.edu

3 Biomedical Informatics Shared Resource, Rutgers Cancer Institute of New Jersey, Rutgers, The State University of New Jersey, New Brunswick, NJ 08903, United States. pengyo@cinj.rutgers.edu

* Corresponding author: welshwj@rwjms.rutgers.edu

Figure S1. Virtual screening cascade is depicted in Figure 1, basically involving QSAR modeling, pharmacophore screening, and shape-based screening.

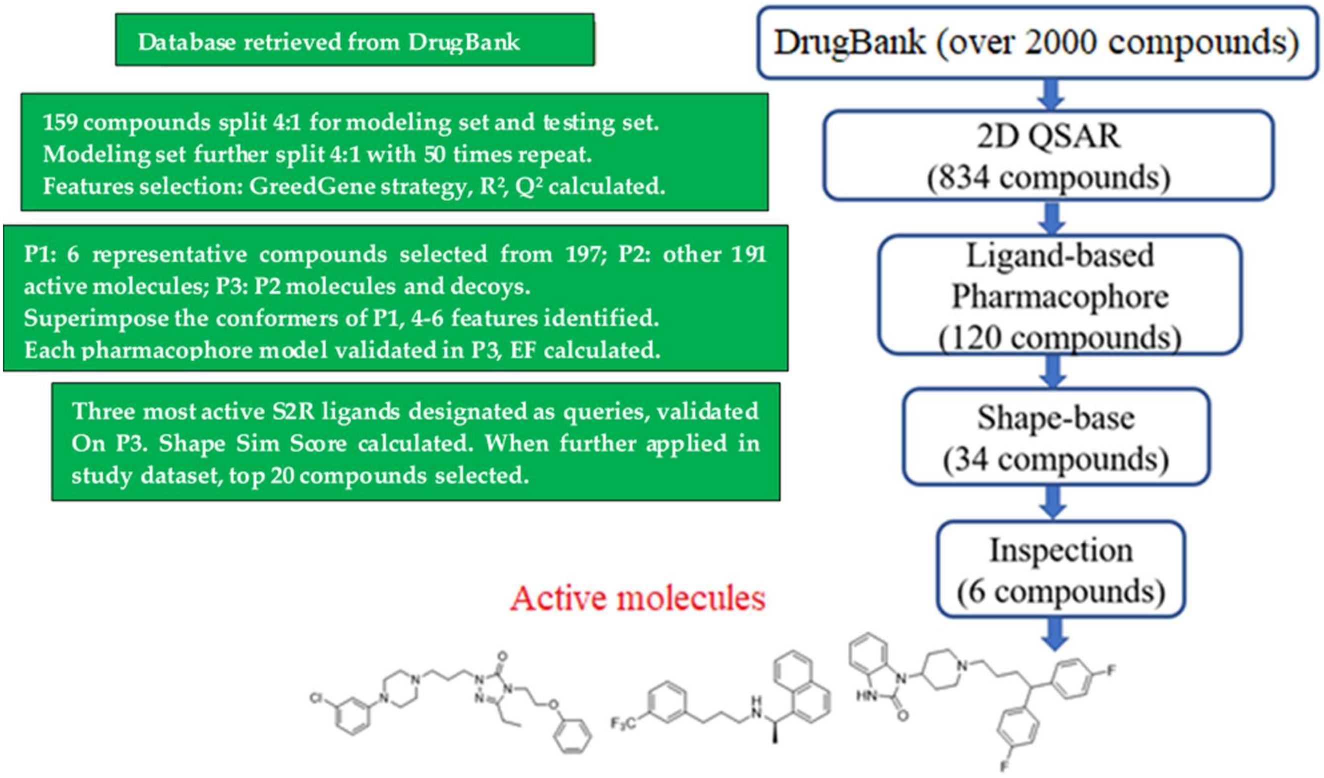

Table S1: Structures and activity data of all S2R ligands pooled from different sources for the present 2D-QSAR studies [1-15]

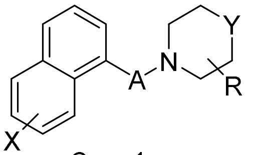
Core 1

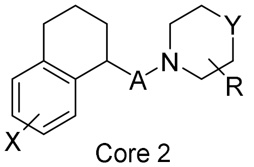
Core 2

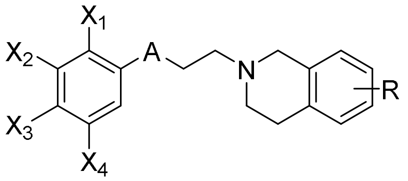
Core 3

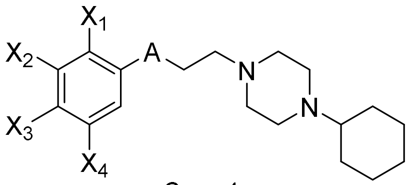
Core 4

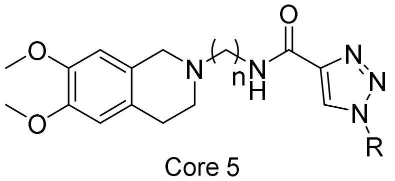
Core 5

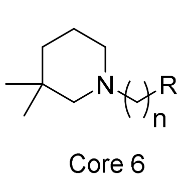
Core 6

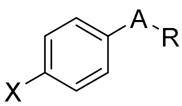
Core 7

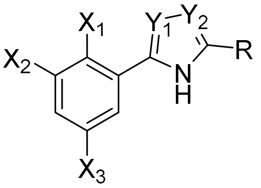
Core 8

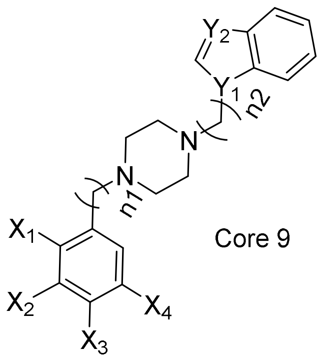
Core 9

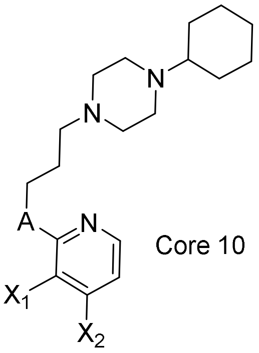
Core 10

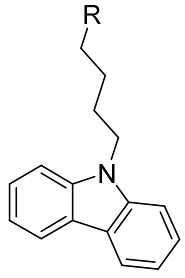
Core 11

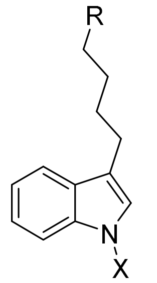
Core 12

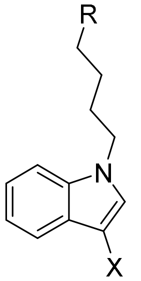
Core 13

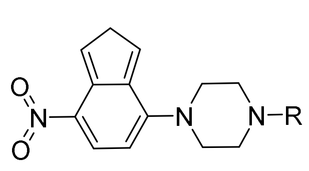
Core 14

Table S2. Summary of the specific scaffold (core), nature of the substituents X, Y, A, etc., values of the experimental and QSAR model-predicted pKi, the Residual (Res.) = Exp. pKi – Pred. pKi, and the identification of the dataset.

No	Core	X	Y	R	A	Exp. pK_i	Pred. pK_i	Res. [#]	Set
1	1	6-OCH_3	NH	4- 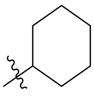	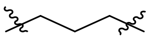	8.03	7.99	-0.04	M
2	1	H	NH	4- 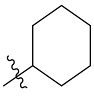	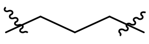	9.16	8.04	-1.12	M
3	1	5-OCH_3	CH_2	H	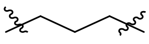	6.76	7.11	0.35	M
4	1	5-OCH_3	CH_2	H	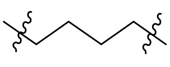	6.82	7.55	0.73	M
5	1	5-OCH_3	CH_2	2,2-di-CH_3	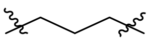	7.04	6.95	-0.09	M
6	1	5-OCH_3	CH_2	3,3-di-CH_3	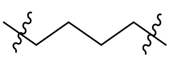	7.17	7.53	0.36	M
7	1	5-OCH_3	CH_2	4,4-di-CH_3	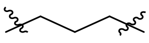	7.58	7.19	-0.39	M
8	1	5-OCH_3	CH_2	4-CH_3	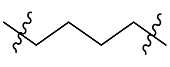	7.75	7.62	-0.13	M
9	1	5-OCH_3	CH_2	4,4-di-CH_3	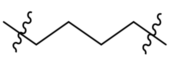	7.75	7.63	-0.12	M
10	1	5-OCH_3	CH_2	2-CH_3	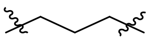	7.06	7.05	-0.01	M
11	1	5-OCH_3	CH_2	2-CH_3	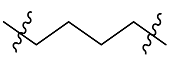	7.19	7.56	0.37	M
12	1	5-OCH_3	CH_2	3-CH_3	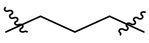	7.22	7.12	-0.1	M
13	1	5-OCH_3	CH_2	2-CH_3	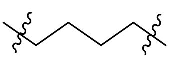	7.31	7.51	0.2	M
14	1	5-OCH_3	CH_2	H	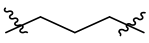	7.58	7.34	-0.24	M
15	1	4-OCH_3	CH_2	3,3-di-CH_3	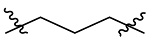	6.17	7.00	0.83	M
16	1	6-OCH_3	CH_2	3,3-di-CH_3	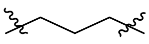	6.19	6.96	0.77	M
17	1	5-OH	CH_2	3,3-di-CH_3	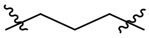	6.22	6.81	0.59	M
18	1	4-OH	CH_2	3,3-di-CH_3	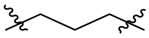	6.39	6.77	0.38	M
19	1	5-OH	NH	4- 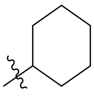	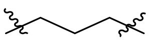	7.58	7.79	0.21	M
20	1	4-OH	NH	4- 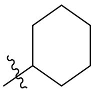	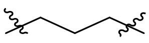	7.93	7.76	-0.17	M
21	1	5-OCH_3	NH	4- 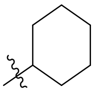	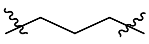	8.04	8.06	0.02	M

22	1	6-OCH$_3$	NH	4- 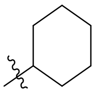	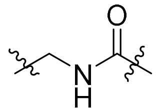	7.63	6.91	-0.72	M
23	1	6-OCH$_3$	NH	4- 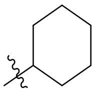	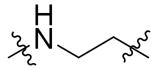	7.73	7.51	-0.22	M
24	1	6-OCH$_3$	NH	4- 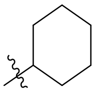	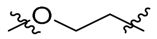	8.62	7.79	-0.83	M
25	2	6-O-CH3	NH	4- 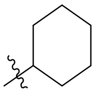	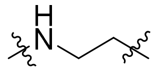	7.84	7.99	0.15	M
26	2	6-O-CH3	NH	4- 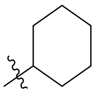	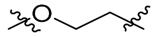	7.95	8.26	0.31	M
27	2	6-O-CH3	NH	4- 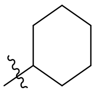	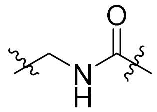	8.23	7.39	-0.84	M
28	2	6-O-CH$_3$	NH	4- 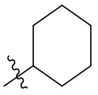	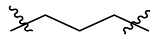	9.31	8.22	-1.09	M
29	1	H	NH	4- 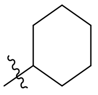	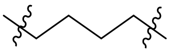	7.52	8.43	0.91	EE
30	1	H	NH	4- 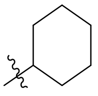	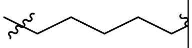	9.24	8.80	-0.44	EE
31	1	5-OCH$_3$	CH$_2$	3,3-di-CH$_3$	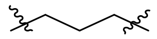	6.62	7.08	0.46	EE
32	1	5-OCH$_3$	CH$_2$	4-CH$_3$	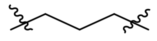	7.41	7.18	-0.23	EE
33	1	5-OCH$_3$	CH$_2$	2,2-di-CH$_3$	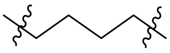	7.54	7.42	-0.12	EE
34	1	4-OCH$_3$	NH	4- 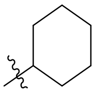	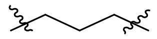	8.08	8.00	-0.08	EE

No	Core	X$_1$	X$_2$	X$_3$	X$_4$	R	A	pK$_i$ exp	pK$_i$ pred	Res.[#]	Set
**35**	3	-OCH$_3$	-OCH$_3$	H	Br	5,6-di-OCH$_3$	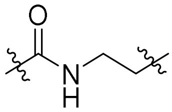	8.09	7.98	-0.11	M
**36**	3	-OCH$_3$	-OCH$_3$	H	Br	H	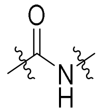	6.14	7.14	1	M
**37**	3	-OCH$_3$	-OCH$_3$	H	Br	5,6-di-OCH$_3$	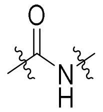	7.79	7.15	-0.64	M

**38**	3	-OCH_3	H	H	-CH_3	5,6-di-OCH_3	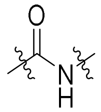	7.88	7.23	-0.65	M
**39**	3	-OCH_3	H	H	Br	5,6-di-OCH_3	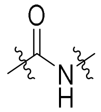	7.91	7.23	-0.68	M
**40**	3	Br	H	-OCH_3	-OCH_3	5,6-di-OCH_3	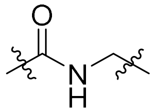	7.29	7.67	0.38	M
**41**	3	-OCH_3	H	H	-CH_3	5,6-di-OCH3	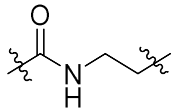	8.06	8.05	-0.01	M
**42**	3	-OCH_3	H	H	-CH_3	5,6-di-OCH3	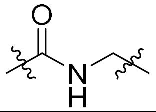	8.58	7.65	-0.93	M
**43**	3	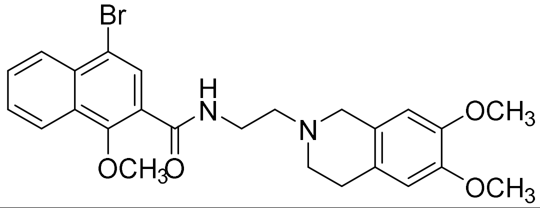						7.67	7.88	0.21	M
**44**	3	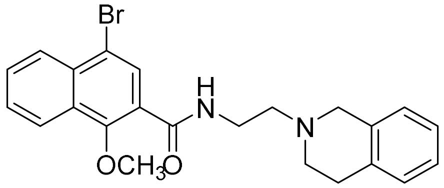						7.32	7.79	0.47	M
**45**	3	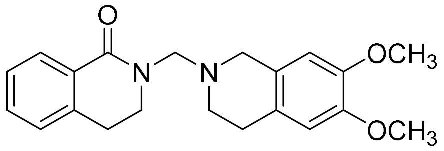						8.32	7.91	-0.41	M
**46**	4	-OCH_3	H	H	-CH_3	-	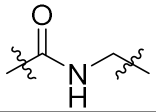	7.58	7.81	0.23	M
**47**	4	-OCH_3	-OCH_3	H	Br	-	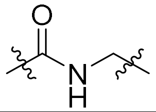	7.67	7.70	0.03	M
**48**	4	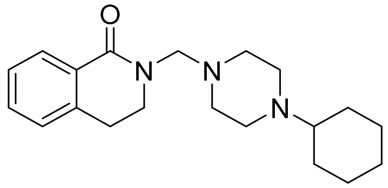						7.58	8.01	0.43	M
**49**	3	-OCH_3	H	H	-CH_3	5,6-di-OCH3	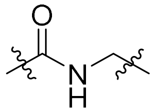	7.99	8.05	0.06	EE
**50**	3	-OCH_3	-OCH_3	H	Br	5,6-di-OCH3	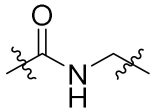	7.89	7.58	-0.31	EE

No	Core	n	R	pK_i exp	pK_i pred	Res.[#]	Set
**51**	5	2	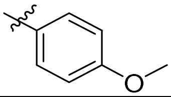	6.39	7.16	0.77	M
**52**	5	2	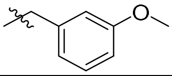	6.67	7.21	0.54	M
**53**	5	2	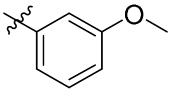	6.74	7.11	0.37	M
**54**	5	2	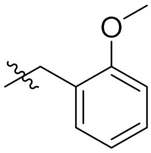	6.81	7.16	0.35	M
**55**	5	2	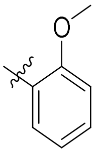	6.82	7.06	0.24	M
**56**	5	2	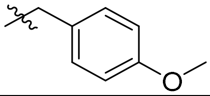	7.03	7.25	0.22	M
**57**	5	4	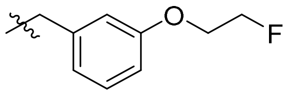	7.21	7.58	0.37	M
**58**	5	4	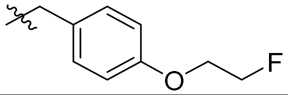	7.32	7.65	0.33	M
**59**	5	4	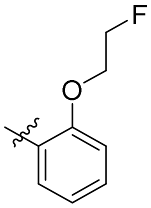	7.62	7.43	-0.19	M
**60**	5	4	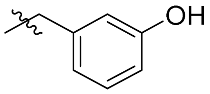	7.65	7.64	-0.01	M
**61**	5	4	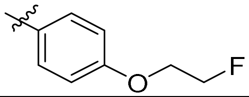	7.66	7.58	-0.08	M
**62**	5	4	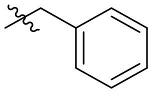	7.91	7.88	-0.03	M
**63**	5	4	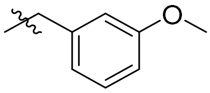	7.97	7.91	-0.06	M
**64**	5	4	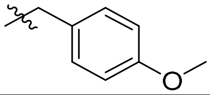	8.30	7.95	-0.35	M
**65**	5	4	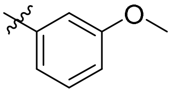	8.52	7.84	-0.68	M
**66**	5	4	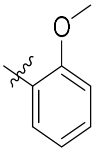	8.82	7.79	-1.03	M

**67**	6	3	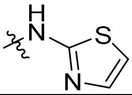	5.48	5.98	0.5	M
**68**	6	3	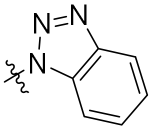	5.70	6.56	0.86	M
**69**	6	1	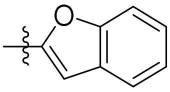	5.74	6.42	0.68	M
**70**	6	3	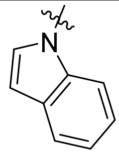	6.71	6.64	-0.07	M
**71**	6	3	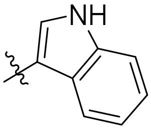	6.96	6.68	-0.28	M
**72**	6	3	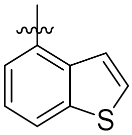	7.20	7.12	-0.08	M
**73**	6	3	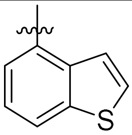	7.37	7.05	-0.32	M
**74**	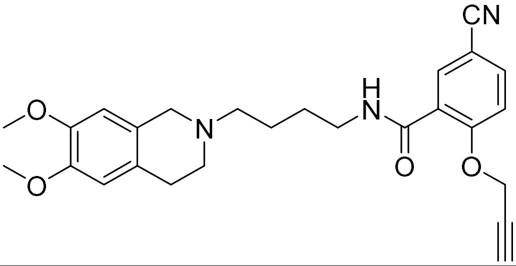			7.91	8.21	0.3	M
**75**	5	4	H	5.99	6.92	0.93	EE
**76**	5	4	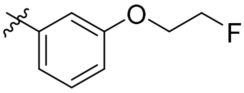	7.6	7.51	-0.09	EE
**77**	5	4	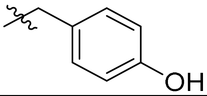	7.94	7.66	-0.28	EE
**78**	5	4	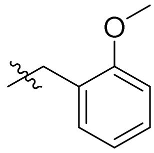	8.01	7.87	-0.14	EE
**79**	5	4	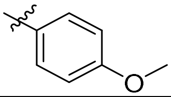	8.26	7.88	-0.38	EE
**80**	6	3	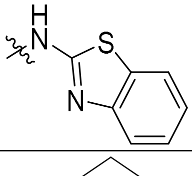	6.13	6.91	0.78	EE
**81**	6	4	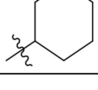	7.71	7.08	-0.63	EE

No	Core	X	A	R	pKi exp	pKi pred	Res. #	Set
**82**	7	F	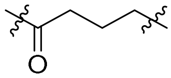	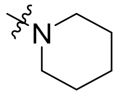	6.49	6.52	0.03	M
**83**	7	-OCH$_2$CH$_2$F	-CH$_2$-	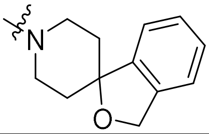	6.56	7.13	0.57	M
**84**	7	Br	-CH$_2$-	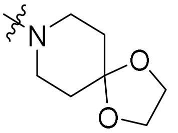	6.63	6.66	0.03	M
**85**	7	I	-CH$_2$-	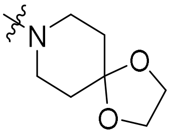	6.63	6.66	0.03	M
**86**	7	F	-CH$_2$-	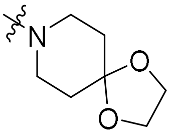	6.79	6.65	-0.14	M
**87**	7	-OCH$_2$CH$_2$F	-CH$_2$-	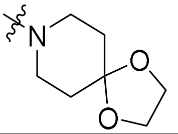	6.79	6.66	-0.13	M
**88**	7	F	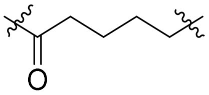	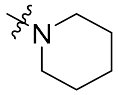	6.97	6.96	-0.01	M
**89**	7	F	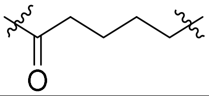	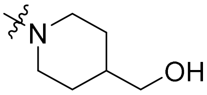	7.05	6.76	-0.29	M
**90**	7	-OCH$_3$	-CH$_2$-	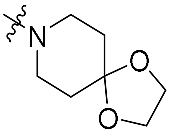	6.28	6.79	0.51	EE
**91**	7	F	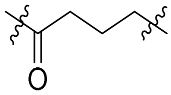	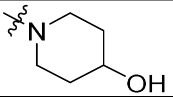	6.30	6.30	0	EE
**92**	7	F	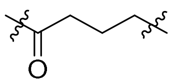	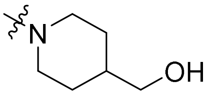	6.46	6.34	-0.12	EE
**93**	7	F	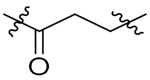	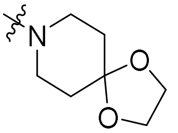	6.51	7.19	0.68	EE
**94**	7	F	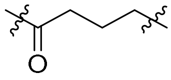	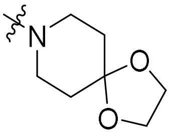	6.75	7.63	0.88	EE

**95**	7	F	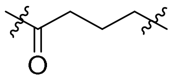	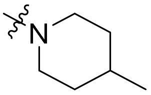	6.86	6.53	-0.33	EE
**96**	7	F	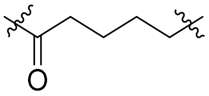	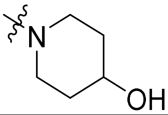	6.93	6.74	-0.19	EE
**97**	7	F	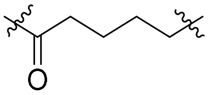	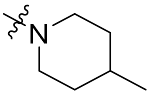	7.64	6.97	-0.67	EE

**No**	**Core**	**X_1**	**X_2**	**X_3**	**Y_1**	**Y_2**	**R**	**pK_i exp**	**pK_i pred**	**Res.** [#]	**Set**
**98**	8	-OCH_3	-OCH_3	Br	N	CH	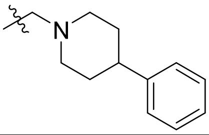	6.39	6.57	0.18	M
**99**	8	-OCH_3	-OCH_3	H	N	O	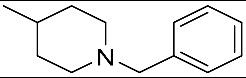	6.60	6.65	0.05	M
**100**	8	-OCH_3	-OCH_3	Br	N	CH	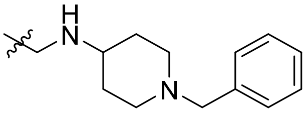	6.85	6.85	0	M
**101**	8	-OCH_3	-OCH_3	Br	N	CH	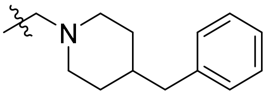	6.95	6.69	-0.26	M
**102**	8	-OCH_3	-OCH_3	Br	CH	CH	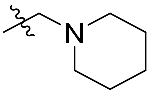	6.29	5.77	-0.52	M
**104**	8	-OCH_3	-OCH_3	Br	CH	CH	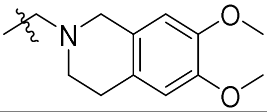	6.34	6.83	0.49	M
**105**	8	-OCH_3	-OCH_3	Br	CH	CH	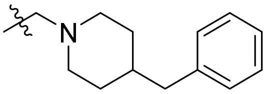	6.45	7.10	0.65	M
**106**	8	-OCH_3	-OCH_3	Br	CH	CH	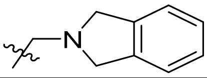	6.51	6.69	0.18	M
**107**	8	-OCH_3	-OCH_3	Br	CH	CH	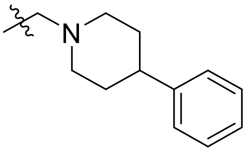	6.66	6.98	0.32	M
**108**	8	-OCH_3	-OCH_3	Br	CH	CH	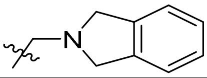	7.06	7.15	0.09	M
**109**	8	-OCH_3	-OCH_3	Br	CH	CH	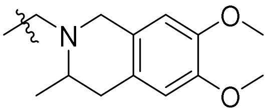	7.28	6.69	-0.59	M
**110**	8	-OCH_3	-OCH_3	Br	CH	CH	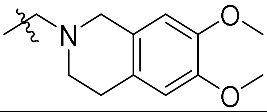	7.59	7.35	-0.24	M

**103**	8	-OCH$_3$	-OCH$_3$	Br	CH	CH	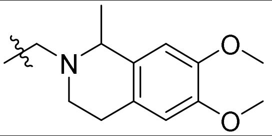	6.30	6.67	0.37	EE

No	Core	X_1	X_2	X_3	X_4	Y_1	Y_2	n_1	n_2	pK_i exp	pK_i pred	Res. #	Set
**111**	9	H	H	F	H	C	N	0	1	6.18	6.86	0.68	M
**112**	9	H	H	H	H	N	C-CH$_3$	2	1	7.25	7.15	-0.1	M
**113**	9	F	H	H	H	N	CH	0	3	8.00	7.58	-0.42	M
**114**	9	F	H	H	F	C	N	1	1	6.38	6.85	0.47	EE
**115**	9	H	F	F	H	C	N	1	1	6.89	6.93	0.04	EE
**116**	9	H	H	F	H	N	CH	0	3	7.70	7.65	-0.05	EE

No	Core	X_1	X_2	A	pK_i exp	pK_i pred	Res. #	Set
**117**	10	H	-OCH$_3$	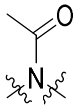	6.61	6.97	0.36	M
**118**	10	H	H	-NH-	7.15	7.06	-0.09	M
**119**	10	H	-OCH$_3$	-NH-	7.22	7.09	-0.13	M
**120**	10	H	-OCH$_3$	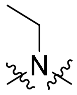	7.52	7.72	0.2	M
**121**	10	H	H	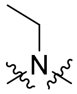	7.83	7.15	-0.68	M
**122**	10	H	H	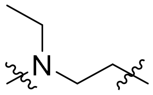	8.17	8.52	0.35	M
**123**	10	H	H	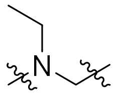	8.45	8.15	-0.3	M
**124**	10	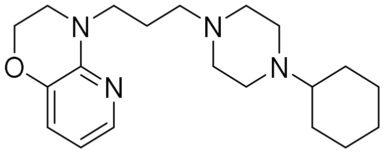			7.73	7.70	-0.03	M
**125**	10	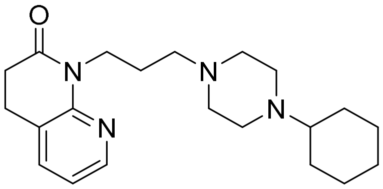			7.79	7.60	-0.19	M

**126**	10	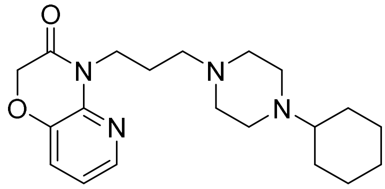			7.80	7.63	-0.17	M
**127**	10	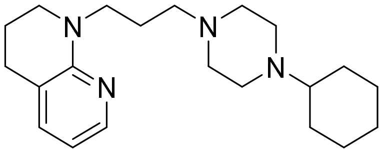			8.01	7.67	-0.34	M
**128**	10	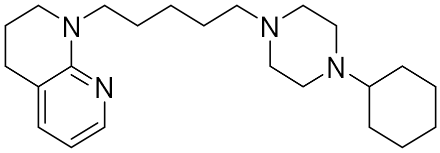			8.19	8.43	0.24	M
**129**	10	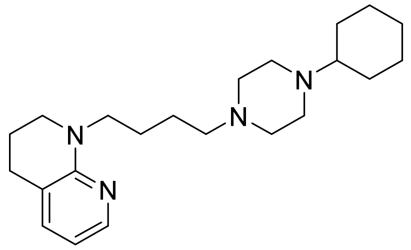			8.79	8.06	-0.73	M
**130**	10	H	H	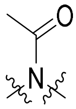	6.51	6.92	0.41	EE

No	Core	R	X	pKi exp	pKi pred	Res. #	Set
**131**	11	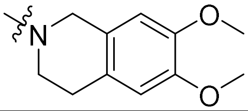	-	10.40	8.62	-1.78	M
**132**	11	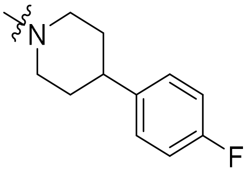	-	7.93	8.75	0.82	M
**133**	11	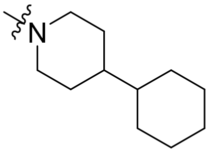	-	8.12	8.71	0.59	M
**134**	11	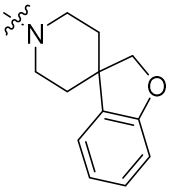	-	8.49	9.02	0.53	M
**135**	12	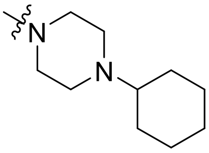	H	8.19	7.99	-0.2	M

**136**	12	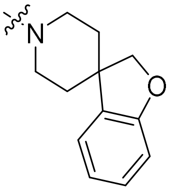	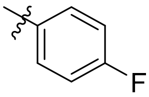	7.90	8.60	0.7	M
**137**	12	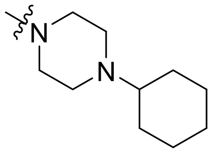	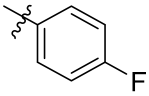	7.54	8.18	0.64	M
**138**	12	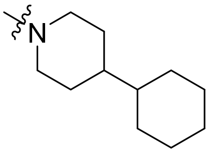	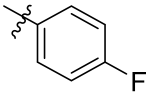	8.14	8.26	0.12	M
**139**	12	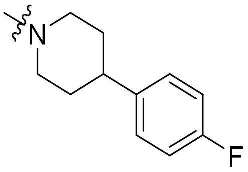	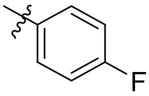	9.57	8.30	-1.27	M
**140**	12	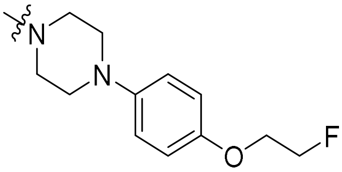	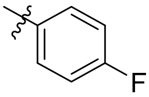	7.17	8.11	0.94	M
**141**	12	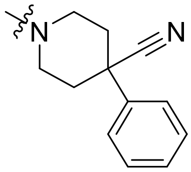	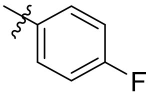	8.52	8.41	-0.11	M
**142**	12	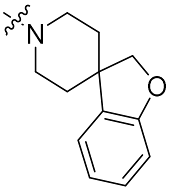	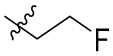	7.36	7.92	0.56	M
**143**	13	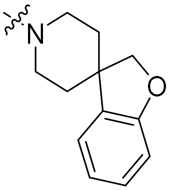	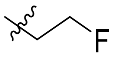	7.53	7.92	0.39	M
**144**	13	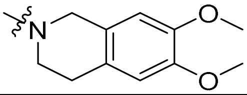	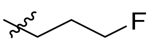	7.55	7.39	-0.16	M
**145**	13	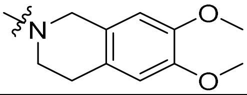	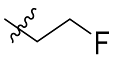	7.56	7.38	-0.18	M
**146**	14	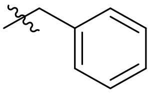	-	5.33	5.86	0.53	M
**147**	14	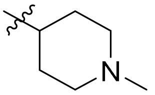	-	6.24	5.65	-0.59	M
**148**	14	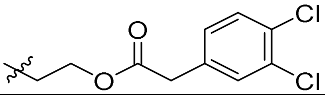	-	6.41	6.57	0.16	M

**149**	14	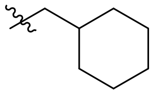	-	6.42	6.09	-0.33	M
**150**	14	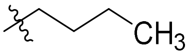	-	6.45	5.55	-0.9	M
**151**	14	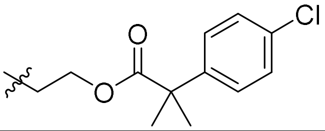	-	6.56	6.40	-0.16	M
**152**	14	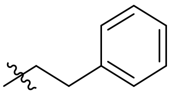	-	6.65	6.26	-0.39	M
**153**	14	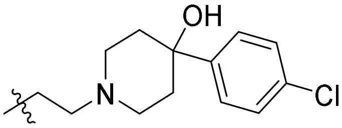	-	6.68	6.77	0.09	M
**154**	14	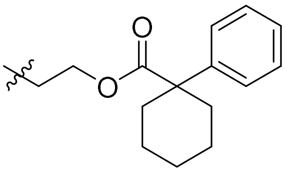	-	7.25	6.80	-0.45	M
**155**	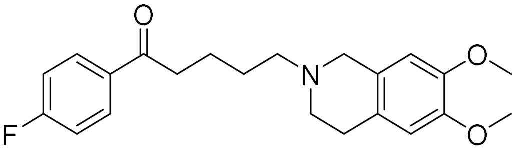			7.91	7.76	-0.15	M
**156**	12	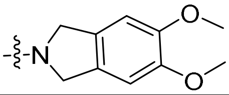	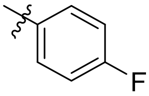	7.27	8.17	0.9	EE
**157**	12	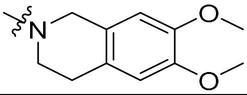	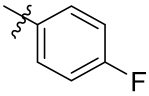	7.31	8.19	0.88	EE
**158**	12	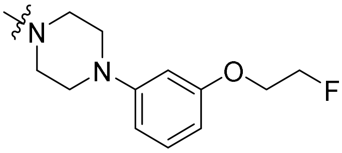	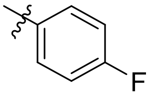	7.32	8.04	0.72	EE
**159**	14	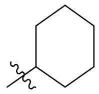	-	6.50	5.66	-0.84	EE

#: Res. (Residual) = pK_i pred - pK_i exp; *: M: Modeling dataset; EE: External evaluation dataset

Table S3: SMILES structure for all S2R ligands used in the present study.

No	SMILES structure
1	C(CCc1c2c([nH]c1)cccc2)C[N+]1CCN(C2CCCCC2)CC1
2	C(C[N+]1CCN(C2CCCCC2)CC1)Cn1c2c(c3c1cccc3)cccc2
3	C(CCn1c2c(c3c1cccc3)cccc2)C[N+]1CCC(C2CCCCC2)CC1
4	Fc1ccc(C2CC[N+](CCCCn3c4c(c5c3cccc5)cccc4)CC2)cc1
5	C(CCn1c2c(c3c1cccc3)cccc2)C[N+]1CCC2(OCc3c2cccc3)CC1
6	O(C)c1c(OC)cc2c(c1)C[N+](CCCCn1c3c(c4c1cccc4)cccc3)CC2
7	Fc1ccc(-n2c3c(c(CCCC[N+]4CCC(C5CCCCC5)CC4)c2)cccc3)cc1
8	Fc1ccc(-n2c3c(c(CCCC[N+]4CCN(C5CCCCC5)CC4)c2)cccc3)cc1
9	Fc1ccc(-n2c3c(c(CCCC[N+]4CCC(c5ccc(F)cc5)CC4)c2)cccc3)cc1
10	Fc1ccc(-n2c3c(c(CCCC[N+]4CCC5(OCc6c5cccc6)CC4)c2)cccc3)cc1
11	Fc1ccc(-n2c3c(c(CCCC[N+]4Cc5c(cc(OC)c(OC)c5)CC4)c2)cccc3)cc1
12	Fc1ccc(-n2c3c(c(CCCC[N+]Cc4ccc(OC)cc4)c2)cccc3)cc1
13	Fc1ccc(-n2c3c(c(CCCC[N+]Cc4cc(OC)ccc4)c2)cccc3)cc1
14	Fc1ccc(-n2c3c(c(CCCC[N+]CCc4cc(OC)ccc4)c2)cccc3)cc1
15	Fc1ccc(-n2c3c(c(CCCC[N+](CCc4ccc(OC)cc4)C)c2)cccc3)cc1
16	Fc1ccc(-n2c3c(c(CCCC[N+](CCc4cc(OC)ccc4)C)c2)cccc3)cc1
17	Fc1ccc(-n2c3c(c(CCCC[N+](CC)Cc4cc(OC)ccc4)c2)cccc3)cc1
18	Fc1ccc(-n2c3c(c(CCCC[N+](CC)Cc4ccc(OC)cc4)c2)cccc3)cc1
19	Fc1ccc(-n2c3c(c(CCCC[N+]Cc4cc(OC)c(OC)cc4)c2)cccc3)cc1
20	Fc1ccc(-n2c3c(c(CCCC[N+]4Cc5c(cc(OC)c(OC)c5)CC4)c2)cccc3)cc1
21	Fc1ccc(-n2c3c(c(CCCC[N+](CCc4cc(OC)c(OC)cc4)C)c2)cccc3)cc1
22	Fc1ccc(-n2c3c(c(CCCC[N+](CC)Cc4cc(OC)c(OC)cc4)c2)cccc3)cc1
23	O(C)c1cc2c(c(CCC[N+]3CCCCC3)ccc2)cc1
24	O(C)c1cc2c(c(CCCC[N+]3CCCCC3)ccc2)cc1
25	O(C)c1cc2c(c(CCC[N+]3[C@H](C)CCCC3)ccc2)cc1
26	O(C)c1cc2c(c(CCC[N+]3C[C@H](C)CCC3)ccc2)cc1
27	O(C)c1cc2c(c(CCC[N+]3CCC(C)CC3)ccc2)cc1
28	O(C)c1cc2c(c(CCC[N+]3C(C)(C)CCCC3)ccc2)cc1
29	O(C)c1cc2c(c(CCCC[N+]3C[C@H](C)CCC3)ccc2)cc1
30	O(C)c1cc2c(c(CCCC[N+]3[C@H](C)CCCC3)ccc2)cc1
31	O(C)c1cc2c(c(CCC[N+]3CCC(C)(C)CC3)ccc2)cc1
32	O(C)c1cc2c(c(CCCC[N+]3CCC(C)CC3)ccc2)cc1
33	O(C)c1cc2c(c(CCCC[N+]3CC(C)(C)CCC3)ccc2)cc1
34	O(C)c1cc2c(c(CCCC[N+]3C(C)(C)CCCC3)ccc2)cc1
35	O(C)c1cc2c(c(CCCC[N+]3CCC(C)(C)CC3)ccc2)cc1
36	Oc1cc2c(c(CCC[N+]3CC(C)(C)CCC3)ccc2)cc1
37	Oc1cc2c(CCC[N+]3CC(C)(C)CCC3)cccc2cc1
38	O(C)c1cc2c(CCC[N+]3CC(C)(C)CCC3)cccc2cc1
39	O(C)c1c2c(c(CCC[N+]3CC(C)(C)CCC3)ccc2)ccc1
40	O(C)c1cc2c(c(CCC[N+]3CC(C)(C)CCC3)ccc2)cc1
41	Oc1cc2c(c(CCC[N+]3CCN(C4CCCCC4)CC3)ccc2)cc1
42	Oc1cc2c(CCC[N+]3CCN(C4CCCCC4)CC3)cccc2cc1
43	O(C)c1c2c(c(CCC[N+]3CCN(C4CCCCC4)CC3)ccc2)ccc1
44	O(C)c1cc2c(c(CCC[N+]3CCN(C4CCCCC4)CC3)ccc2)cc1
45	O(C)c1cc2c(CCC[N+]3CCN(C4CCCCC4)CC3)cccc2cc1
46	O(C)c1c2c(c(NCC[N+]3CCN(C4CCCCC4)CC3)ccc2)ccc1
47	O(CC[N+]1CCN(C2CCCCC2)CC1)c1c2c(c(OC)ccc2)ccc1
48	O(C)c1c2c([C@@H](CCC[N+]3CCN(C4CCCCC4)CC3)CCC2)ccc1
49	O(C)c1c2c([C@H](NCCN3CC[N+](C4CCCCC4)CC3)CCC2)ccc1
50	O(CC[N+]1CCN(C2CCCCC2)CC1)[C@H]1c2c(c(OC)ccc2)CCC1

51	O=C(Nc1c2c(c(OC)ccc2)ccc1)CN1CC[N+](C2CCCCC2)CC1
52	O=C(N[C@H]1c2c(c(OC)ccc2)CCC1)CN1CC[N+](C2CCCCC2)CC1
53	O=[N+]([O-])c1cc2c(cc1)CC[N+](CCCCN1C(=O)c3c(C1=O)cccc3)C2
54	O=[N+]([O-])c1cc2c(cc1)CC[N+](CCCCNC(=O)c1c(OC)c(OC)c(OC)cc1)C2
55	C(CCC1CCCCC1)C[N+]1CC(C)(C)CCC1
56	C(C[N+]1CC(C)(C)CCC1)Cn1c2c(cc1)cccc2
57	C(C[N+]1CC(C)(C)CCC1)Cc1c2c([nH]c1)cccc2
58	C(C[N+]1CC(C)(C)CCC1)Cc1c2c(occ2)ccc1
59	C(C[N+]1CC(C)(C)CCC1)Cc1c2c(scc2)ccc1
60	N(CCC[N+]1CC(C)(C)CCC1)c1sc2c(n1)cccc2
61	O=C(OC1CC2[N+](CCCC[N+])C(C1)CCC2)Nc1c(OC)ccc(C)c1
62	O=C(OC1CC2[N+](CCCCCC[N+])C(C1)CCC2)Nc1c(OC)ccc(C)c1
63	O=C(OC1CC2[N+](CCCCCCCCCC[N+])C(C1)CCC2)Nc1c(OC)ccc(C)c1
64	Brc1cc(C[N+]CCCC[N+]2C3CC(OC(=O)Nc4c(OC)ccc(C)c4)CC2CCC3)ccc1
65	Fc1cc(C[N+]CCCC[N+]2C3CC(OC(=O)Nc4c(OC)ccc(C)c4)CC2CCC3)ccc1
66	Ic1cc(C[N+]CCCC[N+]2C3CC(OC(=O)Nc4c(OC)ccc(C)c4)CC2CCC3)ccc1
67	Brc1ccc(C[N+]CCCC[N+]2C3CC(OC(=O)Nc4c(OC)ccc(C)c4)CC2CCC3)cc1
68	Fc1ccc(C[N+]CCCC[N+]2C3CC(OC(=O)Nc4c(OC)ccc(C)c4)CC2CCC3)cc1
69	Ic1ccc(C[N+]CCCC[N+]2C3CC(OC(=O)Nc4c(OC)ccc(C)c4)CC2CCC3)cc1
70	Brc1cc(C[N+]CCCCCC[N+]2C3CC(OC(=O)Nc4c(OC)ccc(C)c4)CC2CCC3)ccc1
71	Fc1cc(C[N+]CCCCCC[N+]2C3CC(OC(=O)Nc4c(OC)ccc(C)c4)CC2CCC3)ccc1
72	Ic1cc(C[N+]CCCCCC[N+]2C3CC(OC(=O)Nc4c(OC)ccc(C)c4)CC2CCC3)ccc1
73	Brc1ccc(C[N+]CCCCCC[N+]2C3CC(OC(=O)Nc4c(OC)ccc(C)c4)CC2CCC3)cc1
74	Fc1ccc(C[N+]CCCCCC[N+]2C3CC(OC(=O)Nc4c(OC)ccc(C)c4)CC2CCC3)cc1
75	Ic1ccc(C[N+]CCCCCC[N+]2C3CC(OC(=O)Nc4c(OC)ccc(C)c4)CC2CCC3)cc1
76	Clc1ccc(C(=O)NCCCCCC[N+]2C3CC(OC(=O)Nc4c(OC)ccc(C)c4)CC2CCC3)cc1
77	Brc1ccc(C(=O)NCCCCCC[N+]2C3CC(OC(=O)Nc4c(OC)ccc(C)c4)CC2CCC3)cc1
78	Fc1ccc(C(=O)NCCCCCC[N+]2C3CC(OC(=O)Nc4c(OC)ccc(C)c4)CC2CCC3)cc1
79	Ic1ccc(C(=O)NCCCCCC[N+]2C3CC(OC(=O)Nc4c(OC)ccc(C)c4)CC2CCC3)cc1
80	O=C(NCC[N+]1Cc2c(cc(OC)c(OC)c2)CC1)c1c(OC)ccc(C)c1
81	O=C(NCCCC[N+]1Cc2c(cc(OC)c(OC)c2)CC1)c1c(OC)ccc(C)c1
82	Brc1cc(OC)c(OC)c(C(=O)NCC[N+]2Cc3c(cccc3)CC2)c1
83	Brc1c2c(c(OC)c(C(=O)NCC[N+]3Cc4c(cccc4)CC3)c1)cccc2
84	Brc1cc(C(=O)NCC[N+]2Cc3c(cc(OC)c(OC)c3)CC2)c(OC)cc1
85	Brc1cc(OC)c(OC)c(C(=O)NCC[N+]2Cc3c(cc(OC)c(OC)c3)CC2)c1
86	Brc1c2c(c(OC)c(C(=O)NCC[N+]3Cc4c(cc(OC)c(OC)c4)CC3)c1)cccc2
87	Brc1cc(OC)c(OC)c(C(=O)NCCCC[N+]2Cc3c(cc(OC)c(OC)c3)CC2)c1
88	N(CCC[N+]1CCN(C2CCCCC2)CC1)c1ncccc1
89	N(CCC[N+]1CCN(C2CCCCC2)CC1)(CC)c1ncccc1
90	O(C)c1cc(NCCC[N+]2CCN(C3CCCCC3)CC2)ncc1
91	C(C[N+]1CCN(C2CCCCC2)CC1)CN1c2ncccc2CCC1
92	O=C(N(CCC[N+]1CCN(C2CCCCC2)CC1)c1ncccc1)C
93	C(C[N+]1CCN(C2CCCCC2)CC1)CN1c2ncccc2OCC1
94	N(CCCC[N+]1CCN(C2CCCCC2)CC1)(CC)c1ncccc1
95	O=C1N(CCC[N+]2CCN(C3CCCCC3)CC2)c2ncccc2CC1
96	C(CCN1c2ncccc2CCC1)C[N+]1CCN(C2CCCCC2)CC1
97	O=C1N(CCC[N+]2CCN(C3CCCCC3)CC2)c2ncccc2OC1
98	N(CCCCC[N+]1CCN(C2CCCCC2)CC1)(CC)c1ncccc1
99	O(C)c1cc(N(CCC[N+]2CCN(C3CCCCC3)CC2)CC)ncc1
100	C(CC[N+]1CCN(C2CCCCC2)CC1)CCN1c2ncccc2CCC1
101	O=C(N(CCC[N+]1CCN(C2CCCCC2)CC1)c1nccc(OC)c1)C
102	O=C1N(CCC[N+]2CCN(C3CCCCC3)CC2)CCc2c1cccc2
103	O(C)c1c(OC)cc2c(c1)C[N+](CCCN1C(=O)c3c(cccc3)CC1)CC2
104	Brc1cc(OC)c(OC)c(C(=O)NCCC[N+]2CCN(C3CCCCC3)CC2)c1
105	Brc1c(C(=O)NCCC[N+]2Cc3c(cc(OC)c(OC)c3)CC2)cc(OC)c(OC)c1
106	Brc1cc(OC)c(OC)c(C(=O)NCCC[N+]2Cc3c(cc(OC)c(OC)c3)CC2)c1
107	O[C@@H]1C([N+]2C3CC(c4ccccc4)CC2CC3)CCCC1

108	O[C@@H]1C([N+]2C3CC(c4ccccc4)CC2CC3)Cc2c(cccc2)C1
109	Fc1ccc(CN2CC([N+]3C4CC(c5ccccc5)CC3CC4)[C@@H](O)CC2)cc1
110	Ic1cc(CN2CC([N+]3C4CC(c5ccccc5)CC3CC4)[C@@H](O)CC2)ccc1
111	Brc1ccc(C[C@H](O)C[N+]2C3CC(c4ccccc4)CC2CC3)cc1
112	O[C@H](C[N+]1C2CC(c3ccccc3)CC1CC2)c1ccccc1
113	Brc1c([C@H](O)C[N+]2C3CC(c4ccccc4)CC2CC3)cccc1
114	Brc1cc([C@H](O)C[N+]2C3CC(c4ccccc4)CC2CC3)ccc1
115	Brc1ccc([C@H](O)C[N+]2C3CC(c4ccccc4)CC2CC3)cc1
116	Clc1c([C@H](O)C[N+]2C3CC(c4ccccc4)CC2CC3)c(Cl)ccc1
117	Clc1c(Cl)ccc([C@H](O)C[N+]2C3CC(c4ccccc4)CC2CC3)c1
118	Fc1ccc(C(=O)C[N+]2C3CC(c4ccccc4)CC2CC3)cc1
119	Fc1ccc(C(=O)CC[N+]2C3CC(c4ccccc4)CC2CC3)cc1
120	Brc1c2c(c(OC)c(C(=O)N[C@@H]3C[N+](C4C5CCCC4CCC5)CC3)c1)cccc2
121	Brc1c2c(c(OC)c(C(=O)N[C@@H]3C[N+](C4C5CC6CC4CC(C5)C6)CC3)c1)cccc2
122	Brc1c2c(c(OC)c(C(=O)N[C@H]3[N+](C4C5CCCC4CCC5)CCC3)c1)cccc2
123	Brc1c2c(c(OC)c(C(=O)N[C@H]3[N+](C4C5CC6CC4CC(C5)C6)CCC3)c1)cccc2
124	Brc1c2c(c(OC)c(C(=O)NC3CC4[N+](C5CCCCC5)C(C3)CCC4)c1)cccc2
125	Brc1c2c(c(OC)c(C(=O)NC3CC4[N+](C5CCCCCC5)C(C3)CCC4)c1)cccc2
126	Brc1c2c(c(OC)c(C(=O)NC3CC[N+](C4C5CCCC4CCC5)CC3)c1)cccc2
127	Brc1c2c(c(OC)c(C(=O)NC3CC[N+](C4C5CC6CC4CC(C5)C6)CC3)c1)cccc2
128	O=C(NCC[N+]1Cc2c(cc(OC)c(OC)c2)CC1)c1nnn(-c2ccc(OC)cc2)c1
129	O=C(NCC[N+]1Cc2c(cc(OC)c(OC)c2)CC1)c1nnn(-c2cc(OC)ccc2)c1
130	O=C(NCC[N+]1Cc2c(cc(OC)c(OC)c2)CC1)c1nnn(-c2c(OC)cccc2)c1
131	O=C(NCCCC[N+]1Cc2c(cc(OC)c(OC)c2)CC1)c1c(OCC#C)ccc(C#N)c1
132	O=C(NCCCC[N+]1Cc2c(cc(OC)c(OC)c2)CC1)c1nnn(Cc2ccccc2)c1
133	O=C(NCC[N+]1Cc2c(cc(OC)c(OC)c2)CC1)c1nnn(Cc2cc(OC)ccc2)c1
134	O=C(NCC[N+]1Cc2c(cc(OC)c(OC)c2)CC1)c1nnn(Cc2c(OC)cccc2)c1
135	O=C(NCC[N+]1Cc2c(cc(OC)c(OC)c2)CC1)c1nnn(Cc2ccc(OC)cc2)c1
136	O=C(NCCCC[N+]1Cc2c(cc(OC)c(OC)c2)CC1)c1nnn(Cc2cc(O)ccc2)c1
137	O=C(NCCCC[N+]1Cc2c(cc(OC)c(OC)c2)CC1)c1nnn(Cc2ccc(O)cc2)c1
138	O=C(NCCCC[N+]1Cc2c(cc(OC)c(OC)c2)CC1)c1nnn(-c2ccc(OC)cc2)c1
139	O=C(NCCCC[N+]1Cc2c(cc(OC)c(OC)c2)CC1)c1nnn(-c2cc(OC)ccc2)c1
140	O=C(NCCCC[N+]1Cc2c(cc(OC)c(OC)c2)CC1)c1nnn(-c2c(OC)cccc2)c1
141	O=C(NCCCC[N+]1Cc2c(cc(OC)c(OC)c2)CC1)c1nnn(Cc2cc(OC)ccc2)c1
142	O=C(NCCCC[N+]1Cc2c(cc(OC)c(OC)c2)CC1)c1nnn(Cc2c(OC)cccc2)c1
143	O=C(NCCCC[N+]1Cc2c(cc(OC)c(OC)c2)CC1)c1nnn(Cc2ccc(OC)cc2)c1
144	FCCn1nnc(-c2ccc(C(=O)NCCCC[N+]3Cc4c(cc(OC)c(OC)c4)CC3)cc2)c1
145	FCCOc1cc(-n2nnc(C(=O)NCCCC[N+]3Cc4c(cc(OC)c(OC)c4)CC3)c2)ccc1
146	FCCOc1c(-n2nnc(C(=O)NCCCC[N+]3Cc4c(cc(OC)c(OC)c4)CC3)c2)cccc1
147	FCCOc1ccc(-n2nnc(C(=O)NCCCC[N+]3Cc4c(cc(OC)c(OC)c4)CC3)c2)cc1
148	Clc1c(Cl)ccc(CC(=O)OCCN2CCN(c3c4c(c([N+](=O)[O-])cc3)C=CC4)CC2)c1
149	Clc1ccc(C(C(=O)OCCN2CCN(c3c4c(c([N+](=O)[O-])cc3)C=CC4)CC2)(C)C)cc1
150	O=[N+]([O-])c1c2c(c(N3CCN(CCOC(=O)C4(c5ccccc5)CCCCC4)CC3)cc1)CC=C2
151	O=[N+]([O-])c1c2c(c(N3CCN(CC4CCCCC4)CC3)cc1)CC=C2
152	O=[N+]([O-])c1c2c(c(N3CCN(CCCC)CC3)cc1)CC=C2
153	Clc1cc(C2(O)CCN(CCN3CCN(c4c5c(c([N+](=O)[O-])cc4)C=CC5)CC3)CC2)ccc1
154	O=[N+]([O-])c1c2c(c(N3CCN(CCc4ccccc4)CC3)cc1)CC=C2
155	O=[N+]([O-])c1c2c(c(N3CCN(C4CCCCC4)CC3)cc1)CC=C2
156	Fc1cc2n(-c3ccc(F)cc3)cc(CCCCN3Cc4c(cc(OC)c(OC)c4)CC3)c2cc1
157	Fc1ccc(-n2c3c(c(CCCCN4Cc5c(cc(OC)c(OC)c5)C4)c2)cccc3)cc1
158	Fc1ccc(-n2c3c(c(CCCCN4Cc5c(cc(OC)c(OC)c5)CC4)c2)cccc3)cc1
159	FCCOc1cc(N2CCN(CCCCc3c4c(n(-c5ccc(F)cc5)c3)cccc4)CC2)ccc1

References cited in Table S1.

1. Xie, F.; Kniess, T.; Neuber, C.; Deuther-Conrad, W.; Mamat, C.; Lieberman, B. P.; Liu, B.; Mach, R. H.; Brust, P.; Steinbach, J.; Pietzsch, J.; Jia, H., Novel indole-based sigma-2 receptor ligands: synthesis, structure–affinity relationship and antiproliferative activity. *MedChemComm* **2015**, 6, 1093-1103.
2. Xie, F.; Bergmann, R.; Kniess, T.; Deuther-Conrad, W.; Mamat, C.; Neuber, C.; Liu, B.; Steinbach, J.; Brust, P.; Pietzsch, J.; Jia, H., (18)F-Labeled 1,4-Dioxa-8-azaspiro[4.5]decane Derivative: Synthesis and Biological Evaluation of a sigma1 Receptor Radioligand with Low Lipophilicity as Potent Tumor Imaging Agent. *J Med Chem* **2015**, 58, 5395-407.
3. Schininà, B.; Martorana, A.; Colabufo, N. A.; Contino, M.; Niso, M.; Perrone, M. G.; De Guidi, G.; Catalfo, A.; Rappazzo, G.; Zuccarello, E.; Prezzavento, O.; Amata, E.; Rescifina, A.; Marrazzo, A., 4-Nitro-2,1,3-benzoxadiazole derivatives as potential fluorescent sigma receptor probes. *RSC Advances* **2015**, 5, 47108-47116.
4. Bai, S.; Li, S.; Xu, J.; Peng, X.; Sai, K.; Chu, W.; Tu, Z.; Zeng, C.; Mach, R. H., Synthesis and structure-activity relationship studies of conformationally flexible tetrahydroisoquinolinyl triazole carboxamide and triazole substituted benzamide analogues as sigma2 receptor ligands. *J Med Chem* **2014**, 57, 4239-51.
5. Niso, M.; Abate, C.; Contino, M.; Ferorelli, S.; Azzariti, A.; Perrone, R.; Colabufo, N. A.; Berardi, F., Sigma-2 receptor agonists as possible antitumor agents in resistant tumors: hints for collateral sensitivity. *ChemMedChem* **2013**, 8, 2026-35.
6. Abate, C.; Ferorelli, S.; Niso, M.; Lovicario, C.; Infantino, V.; Convertini, P.; Perrone, R.; Berardi, F., 2-Aminopyridine derivatives as potential sigma(2) receptor antagonists. *ChemMedChem* **2012**, 7, 1847-57.
7. Yarim, M.; Koksal, M.; Schepmann, D.; Wunsch, B., Synthesis and in vitro Evaluation of Novel Indole-Based Sigma Receptors Ligands. *Chem Biol Drug Des* **2011**, 78, 869-75.
8. Ferorelli, S.; Abate, C.; Pedone, M. P.; Colabufo, N. A.; Contino, M.; Perrone, R.; Berardi, F., Synthesis and binding assays of novel 3,3-dimethylpiperidine derivatives with various lipophilicities as sigma(1) receptor ligands. *Bioorg Med Chem* **2011**, 19, 7612-22.
9. Abate, C.; Niso, M.; Lacivita, E.; Mosier, P. D.; Toscano, A.; Perrone, R., Analogues of sigma receptor ligand 1-cyclohexyl-4-[3-(5-methoxy-1,2,3,4-tetrahydronaphthalen-1-yl)propyl]piperazine (PB28) with added polar functionality and reduced lipophilicity for potential use as positron emission tomography radiotracers. *J Med Chem* **2011**, 54, 1022-32.
10. Abate, C.; Ferorelli, S.; Contino, M.; Marottoli, R.; Colabufo, N. A.; Perrone, R.; Berardi, F., Arylamides hybrids of two high-affinity sigma2 receptor ligands as tools for the development of PET radiotracers. *Eur J Med Chem* **2011**, 46, 4733-41.

11. Ferorelli, S.; Abate, C.; Colabufo, N. A.; Niso, M.; Inglese, C.; Berardi, F.; Perrone, R., Design and evaluation of naphthol- and carbazole-containing fluorescent sigma ligands as potential probes for receptor binding studies. *J Med Chem* **2007**, 50, 4648-55.
12. Berardi, F.; Ferorelli, S.; Abate, C.; Pedone, M. P.; Colabufo, N. A.; Contino, M.; Perrone, R., Methyl substitution on the piperidine ring of N-[omega-(6-methoxynaphthalen-1-yl)alkyl] derivatives as a probe for selective binding and activity at the sigma(1) receptor. *J Med Chem* **2005**, 48, 8237-44.
13. Mach, R. H.; Huang, Y.; Freeman, R. A.; Wu, L.; Vangveravong, S.; Luedtke, R. R., Conformationally-flexible benzamide analogues as dopamine D3 and sigma 2 receptor ligands. *Bioorg Med Chem Lett* **2004**, 14, 195-202.
14. Berardi, F.; Ferorelli, S.; Abate, C.; Colabufo, N. A.; Contino, M.; Perrone, R.; Tortorella, V., 4-(tetralin-1-yl)- and 4-(naphthalen-1-yl)alkyl derivatives of 1-cyclohexylpiperazine as sigma receptor ligands with agonist sigma2 activity. *J Med Chem* **2004**, 47, 2308-17.
15. Mach, R. H.; Huang, Y.; Freeman, R. A.; Wu, L.; Blair, S.; Luedtke, R. R., Synthesis of 2-(5-Bromo-2,3-dimethoxyphenyl)-5-(aminomethyl)-1H-pyrrole analogues and their binding affinities for dopamine D2, D3, and D4 receptors. *Bioorganic & Medicinal Chemistry* **2003**, 11, 225-233.
